# More Challenging Diets Sustain Feeding Performance: Applications Toward the Captive Rearing of Wildlife

**DOI:** 10.1093/iob/obab030

**Published:** 2021-11-22

**Authors:** D Rex Mitchell, Stephen Wroe, Matthew J Ravosa, Rachel A Menegaz

**Affiliations:** Center for Anatomical Sciences, University of North Texas Health Science Center, Fort Worth, TX 76107, USA; College of Science and Engineering, Flinders University, Adelaide, SA 5042, Australia; School of Environmental and Rural Science, University of New England, Armidale, NSW 2351, Australia; Departments of Biological Sciences, Aerospace and Mechanical Engineering, and Anthropology, University of Notre Dame, Notre Dame, IN 46556, USA; Center for Anatomical Sciences, University of North Texas Health Science Center, Fort Worth, TX 76107, USA

## Abstract

The rescue and rehabilitation of young fauna is of substantial importance to conservation. However, it has been suggested that incongruous diets offered in captive environments may alter craniofacial morphology and hinder the success of reintroduced animals. Despite these claims, to what extent dietary variation throughout ontogeny impacts intrapopulation cranial biomechanics has not yet been tested. Here, finite element models were generated from the adult crania of 40 rats (*n* = 10 per group) that were reared on 4 different diet regimes and stress magnitudes compared during incisor bite simulations. The diets consisted of (1) exclusively hard pellets from weaning, (2) exclusively soft ground pellet meal from weaning, (3) a juvenile switch from pellets to meal, and (4) a juvenile switch from meal to pellets. We hypothesized that a diet of exclusively soft meal would result in the weakest adult skulls, represented by significantly greater stress magnitudes at the muzzle, palate, and zygomatic arch. Our hypothesis was supported at the muzzle and palate, indicating that a diet limited to soft food inhibits bone deposition throughout ontogeny. This finding presents a strong case for a more variable and challenging diet during development. However, rather than the “soft” diet group resulting in the weakest zygomatic arch as predicted, this region instead showed the highest stress among rats that switched as juveniles from hard pellets to soft meal. We attribute this to a potential reduction in number and activity of osteoblasts, as demonstrated in studies of sudden and prolonged disuse of bone. A shift to softer foods in captivity, during rehabilitation after injury in the wild for example, can therefore be detrimental to healthy development of the skull in some growing animals, potentially increasing the risk of injury and impacting the ability to access full ranges of wild foods upon release. We suggest captive diet plans consider not just nutritional requirements but also food mechanical properties when rearing wildlife to adulthood for reintroduction.

## Introduction

Captive rearing and rehabilitation of wildlife represents an important aspect of conservation efforts. Each year, many wild animals are rescued, be it due to injury, illness, abandonment, or parental mortality (Vogelnest 2008). For example, Tribe and Brown (2000) identify over 200,000 cases of wildlife taken to wildlife careers over a five-year period across three Australian states, while Wildlife Rescue & Rehabilitation, based in Texas, reports receiving approximately 10,000 native wild animals every year (Wildlife Rescue 2016). The ultimate goal in these rescue efforts is to rehabilitate and release these animals once they are old enough and fit enough to fend for themselves. Yet, there can be stark contrasts between captive and wild diet regimes (Glatt et al. 2008). Captive diets can consist of softer, processed, or pre-portioned foods that meet the nutritional requirements of the animals but differ in, or lack altogether, the structural complexity or procurement effort associated with wild foods. For example, carnivorous mammals may be fed minced meat that lacks any mechanical variation usually offered by bones, cartilage, tendon, and hide (Hartstone-Rose et al. 2014) or herbivores may be fed small pellets (e.g., Dierenfield 1997) that bypass the need for animals to crop food with their incisors and that can be passed directly to the cheek teeth. There is concern that incongruous diets offered in captivity may negatively impact the success of released animals (Wisely et al. 2005; Siciliano-Martina et al. 2021a).

Populations of many species in captivity can exhibit different skull dimensions to wild populations. O'Regan and Kitchener (2005) noted that most differences in cranial morphology are often centered around the feeding apparatus. These can include zygomatic arch breadth, snout length and breadth, facial height, and mandibular proportions (e.g., Hollister 1917; Bouvier and Hylander 1981; Corruccini and Beecher 1982, 1984; Geiser and Ferguson 2001; O'Regan 2001; Zuccarelli 2004; Hartstone-Rose et al. 2014; Curtis et al. 2018; Siciliano-Martina et al. 2021a). Contrasting morphology observed in captivity has therefore often been attributed to the different material properties (stiffness/toughness) of captive diet constituents and the biomechanical requirements for their processing (e.g., O'Regan and Kitchener 2005; Hartstone-Rose et al. 2014; Curtis et al. 2018; Selvey 2018; Neaux et al. 2021). However, differences from wild populations are often quantified after multiple generations of captivity, which leaves room for other potential drivers of morphological diversity, such as size-related differences (static, ontogenetic, and evolutionary allometry) (Klingenberg and Zimmerman 1992; Wisely et al. 2005), random walk, founder effects, or relaxed selection (McPhee 2004; Siciliano-Martina et al. 2021b). The extent to which these trends between skull morphology and diet apply to wild-captured young within a single generation of captivity remains largely unexplored.

Despite biomechanical inferences being drawn from results of morphometric tests on cranial shape (e.g., linear measurements, geometric morphometrics; Zuccarelli 2004; Hartstone-Rose et al. 2014; Curtis et al. 2018; Siciliano-Martina et al. 2021a, 2021b), the impact of diet on intrapopulation cranial biomechanics itself has received little experimental attention (but see Smith et al. 2015; Mitchell et al. 2020; Brachetta-Aporta and Toro-Ibacache 2021 as intraspecific examples). Lieberman et al. (2004) tested the influence of diet on the facial growth and *in vivo* strains (bone deformation) of rock hyraxes (*Procavia capensis*). They found greater strain magnitudes in the crania of individuals fed softer foods. However, the test subjects for their study only comprised eight individuals and were already juveniles (5–6 months old) at the start of the study, potentially missing the early postweaning period of growth during which mammals typically adopt adult-like jaw adductor muscle and feeding actions (Ravosa et al. 2008a, 2008b, 2016). Thus, the impact that different foods with contrasting material properties, processed from weaning to adulthood, have on the ability of an adult to bite effectively remains elusive but is relevant to questions related to designing feeding protocols for rescued altricial wildlife. Here, we specifically address whether the material properties of food alone can affect the biomechanical performance of the cranium within a single generation raised from weaning to adulthood.

Performance refers to the success that a given phenotype has when accomplishing a particular task (see Koehl 1996). A methodology that allows us to assess the biomechanical performance of bone is finite element analysis. This is a computational engineering tool that involves simulating behaviors or actions of interest on digital models rendered from scanned specimens (Richmond et al. 2005; Rayfield 2007; Panagiotopoulou 2009; Bright 2014). Relative performance metrics such as mechanical efficiency (output force/total applied muscle force), stress (force per unit area), and strain (Δlength/initial length) can be obtained from modeled skulls and are often attributed to known or predicted diets and feeding behaviors across the species examined (e.g., Wroe et al. 2007, 2013; Porro et al. 2011; Ross et al. 2011; Cox et al. 2012; Oldfield et al. 2012; Smith et al. 2015; Tseng and Flynn 2015; Godinho et al. 2018; Lautenschlager et al. 2018; Ledogar et al. 2018; Mitchell et al. 2018; Panagiotopoulou et al. 2020). In order to highlight potential biomechanical deficits introduced by soft diets in captive-reared fauna, we employ the finite element method here to test the influence that contrasting food material properties have on bone deposition, and resulting biting performance, in a single generation of animals raised from weaning.

Growing bones are likely to be particularly susceptible to more extreme changes in bone physiology imparted by alternative degrees of forces applied (loading) (Carter 1984; Hinton and McNamara 1984; Bouvier 1988; Rubin et al. 1992; Pearson and Lieberman 2004; but see Scott et al. 2014). This suggests that controlled manipulation of diet throughout the entirety of postweaning ontogeny could reasonably be expected to maximize differences in biomechanical performance in adult crania (Lieberman et al. 2004). However, wildlife young are often rescued at different stages of development, from weaning through to juvenile stages and beyond. This means that switches in food properties are likely to occur at various stages of development when captive diet regimes are introduced during rehabilitation. For this reason, we aimed to test the impact of food material properties from weaning to adulthood and also diet switches that may occur in mid-development.

We examined the crania of rats fed contrasting diets. Four groups of rats (*n* = 10 per group) were fed standard industry rodent feed in either a mechanically challenging “hard” pellet form or as “soft” ground pellet meal. These diets were consistent in nutritional content and only differed in the way they are processed in the oral cavity. In contrast to ground pellets that likely require minimal oral breakdown (including both the initial reduction of particle size via incision and further breakdown during mastication), intact pellets require greater initial incisal biting forces and greater subsequent cyclical loading during repeated grinding at the molars. This results in longer feeding cycles. One cohort was fed a consistent diet of pellets from weaning to adulthood; another was fed a consistent diet of meal. The two remaining groups had their diet switched at the juvenile stage. In the context of rearing wildlife, abandoned or orphaned animals rescued at a weaning stage are represented by the two diets consisting of exclusively hard pellets or soft meal. The two diets that switched at the juvenile stage represent diet switches that may occur if juveniles are rescued from the wild, due to injury or disease, and are reared to adulthood in captivity on a mechanically different diet to their wild selection.

Our specific aims revolve around the highly dynamic nature of bone as a living tissue. Its ability to adapt a genetically predetermined shape to mechanical loading over time has been well established for over 100 years (Roux 1881; Wolff 1892; Frost 1994; Pearson and Lieberman 2004). This is primarily driven by strain (bone bending, or “deformation”) experienced during the application of forces (Carter 1984; Rubin and Lanyon 1985; Bentolila et al. 1998; Hammer 2015). During vertebrate growth, a dominant feature of bone physiology is “modeling,” during which bone increases in size (both longitudinally and radially) by the addition of bone mass. After skeletal maturity, “remodeling” becomes the more dominant feature of bone physiology wherein mature bone is replaced in part to facilitate the repair of crack formation. These mechanisms have been demonstrated across such disparate taxa as birds, marsupials, rodents, and primates (e.g., Bouvier and Hylander 1981; Mosley et al. 1997; Mosley and Lanyon 1998; Lieberman and Crompton 1998; Judex and Zernicke 2000). The vertebrate cranium undergoes deformation during biting and chewing, as reaction forces along the teeth and at the temporomandibular joints arise in response to applied forces from the muscles of mastication and are transmitted and dissipated throughout the cranium (Wang et al. 2008). Accordingly, bone remodeling has been observed in response to processing foods of different mechanical resistance (e.g., Bouvier and Hylander 1981, 1996; Kiliaridis et al. 1985; Kopher and Mao 2003; Organ et al. 2006; Ravosa et al. 2016; Menegaz and Ravosa 2017; Terhune et al. 2020; Lad et al. 2021). Higher peak strains (during hard biting) or cyclical strains (during repeated grinding) generated by the consumption of more resistant foods are therefore expected to facilitate the deposition of more bone during ontogeny.

**Fig. 1:**
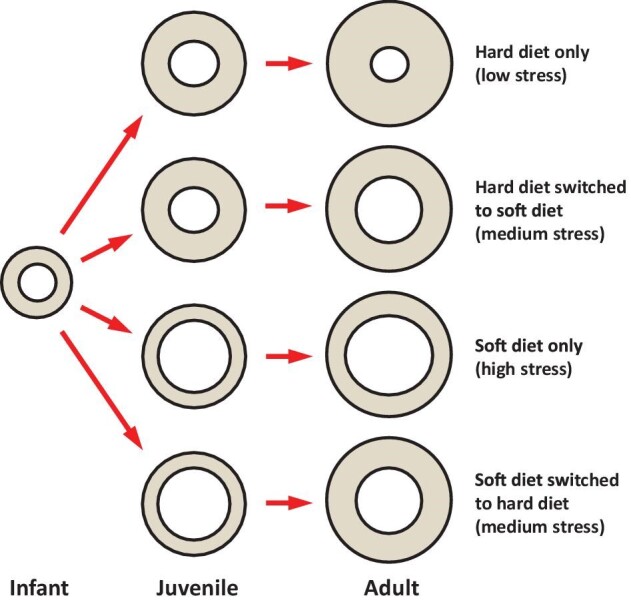
Schematic representation of hypothesized bone growth for each diet group through ontogeny (modified from Ruff et al. 1994). Diets of mechanically challenging foods will increase bone deposition. Thicker bone will accommodate higher peak and cyclical forces and reduce deformation. Therefore, we expect higher stress magnitudes throughout the crania raised on “soft” diets when subjected to equivalent muscle forces in silico.

We hypothesized that finite element models of rats fed exclusively “soft” food would demonstrate weaker biomechanical performance during an incisor bite than those fed “harder” food, evidenced by greater estimated stresses across the cranium during incisal bite simulations. Stress refers to the amount of force per unit area experienced at a specific location of an object during an action. In the context of this study, stress is defined as force per unit of bone, so an increase in bone mass at a specific region of the cranium will decrease the amount of stress experienced at that region for a given amount of force (see Mitchell 2019). We expected group differences in stress magnitudes to be most obvious in cranial regions that demonstrate high levels of strain when biting, namely the superior muzzle base and inferior aspect of the zygomatic arch (Hylander and Johnson 1997; Franks et al. 2016, 2017; Mitchell et al. 2020), but also along the narrow strip of bone between the large anterior palatine foramina, as this is a particularly long and thin stretch of bone in rodents (see Missagia and Perini 2018). In addition, we also anticipated that animals fed diets that switched between “hard” and “soft” foods as juveniles would demonstrate stress levels within the extremes determined by the exclusively hard and soft food groups (Fig. 1).

## Materials and methods

This study uses existing microcomputed tomography (μCT) scans (Menegaz 2013; Menegaz and Ravosa 2017). All procedures were conducted in accordance with a University of Missouri Institutional Animal Care and Use Committee approved protocol (Protocol number: 6622).

The Sprague-Dawley rats modeled here (*Rattus norvegicus*, RRID:RGD_5508397) (Berkenhout, 1769) were obtained as 22-day-old weanlings from Harlan Laboratories (Haslett, MI). These rats were all from the same colony, were the same age, all male, reared in the same captive environment, and received the same nutritional requirements. This sample therefore effectively controls for any potentially confounding signals that may arise from genetics, age differences, sex differences, environmental variables, and nutritional variation. The rats were randomly sorted into four distinct dietary cohorts and reared on their allocated diet regimes for 12 weeks. This time frame encompasses the developmental range of skeletal maturity in these rats (Roach et al. 2003). All groups were fed LabDiet 5001 Rodent Diet (PMI Nutrition International, St. Louis, MO). The feeding protocol is detailed in Table 1 and, for simplicity, we henceforth refer to these diet groups as hard, hard:soft, soft, and soft:hard. With a mean Young's modulus of 13.61 MPa, a mean hardness of 7.25, and a mean toughness of 3325.12 J m^–2^, the pellets fall within the range of toughness and elasticity of foods commonly consumed by wild mammals. They are mechanically most similar to underground storage organs, such as wild roots and tubers (Menegaz 2013). Conversely, based on our *in vivo* observations, the consumption of the meal diet requires little-to-no oral processing before swallowing.

**Table 1:** Experimental diet groups

Cohort (*n* = 10 per group)	Weaning to adolescence (week 4–week 10)	Adolescence to adult (week 10–week 16)
1. Hard diet	Pellet	Pellet
2. Hard:soft diet	Pellet	Meal
3. Soft diet	Meal	Meal
4. Soft:hard diet	Meal	Pellet

We used *in vivo* μCT scans produced at 16 weeks of age. The rat heads were imaged using a Siemens Inveon Micro-SPECT/CT unit (Siemens Pre-Clinical Solutions, Knoxville, TN, USA), operated at 80 kV and 500 mA, with reconstruction using 0.126 mm^3^ voxels for all individuals.

Recently, Tseng (2021) found that limited numbers of finite element models may result in elevated correlations and false positives. At this scale of relatedness between individuals, we expected a high degree of consistency in stress and strain distributions (Smith et al. 2015; Brachetta-Aporta and Toro-Ibacache 2021), with many instances of overlap between diet groups. This prompted us to generate the most thorough sample of finite element models compiled to date by modeling all 40 individuals, in order to tease apart any fine-scale intrapopulation differences in performance.

3D surface meshes of the crania and mandibles were created from the μCT data in Mimics (Materialise v. 21). For each model, the cranium was centered and then oriented such that the vertical axis aligned with the principal axis of the incisors. The mandible was then positioned for incisor contact to simulate a rodent biting or gnawing action.

Cranial meshes were then exported and converted to finite element models (volume meshes) using 3-Matic (Materialise v. 13.0). Each model consisted of approximately 1.7 million 3D tetrahedral elements (bricks). Models were then imported into Strand7 (v. 2.4.4) finite element software. The bricks were assigned homogeneous, isotropic material properties of cortical bone from a rat femur aged 18 weeks (Young's modulus: E ∼ 15 GPa; Poisson's ratio: *v* = 0.3) (Vanleene et al. 2008). Bone properties are known to be variable across the skull (Franks et al. 2017); however, our hypothesis was primarily concerned with the comparisons of the gross bone architecture of entire crania between diet groups. Therefore, homogeneous and isotropic material properties were considered acceptable to assess the relationship between cranial morphology and performance (Strait et al. 2010; Walmsley et al. 2013; Fitton et al. 2015). Our results should be considered in a relative context and not as actual vivo stress magnitudes.

We modeled all jaw adductor muscles of a rat: the temporalis, superficial masseter, deep masseter, zygomaticomandibularis, intraorbital portion of the zygomaticomandibularis, internal pterygoids and external pterygoids. Masticatory muscle origins and insertions following Hiiemäe and Houston (1971) were allocated to the modeled crania and mandible surface meshes. The masticatory muscle forces of a rat (Cox et al. 2012) were applied to the cranial plates using BoneLoad (Grosse et al. 2007). This software orients the forces from the cranial muscle origins to the centroids of their respective insertions, following the curvature of the bone. The muscle forces were initially applied to a randomly selected individual as a reference and the muscle forces for all other individuals were scaled to cranial volume using a 2/3 power rule (Ledogar et al. 2016) (Table S1). The loaded plates were imported into Strand7 and zipped to the nodes of their corresponding elements. A single node on the tip of each incisor was restrained against translation in the vertical axis and a single node at each temporomandibular joint was restrained against translation for all axes. This configuration simulates a bilateral incisor bite (Mitchell et al. 2018; Brachetta-Aporta and Toro-Ibacache 2021).

**Fig. 2:**
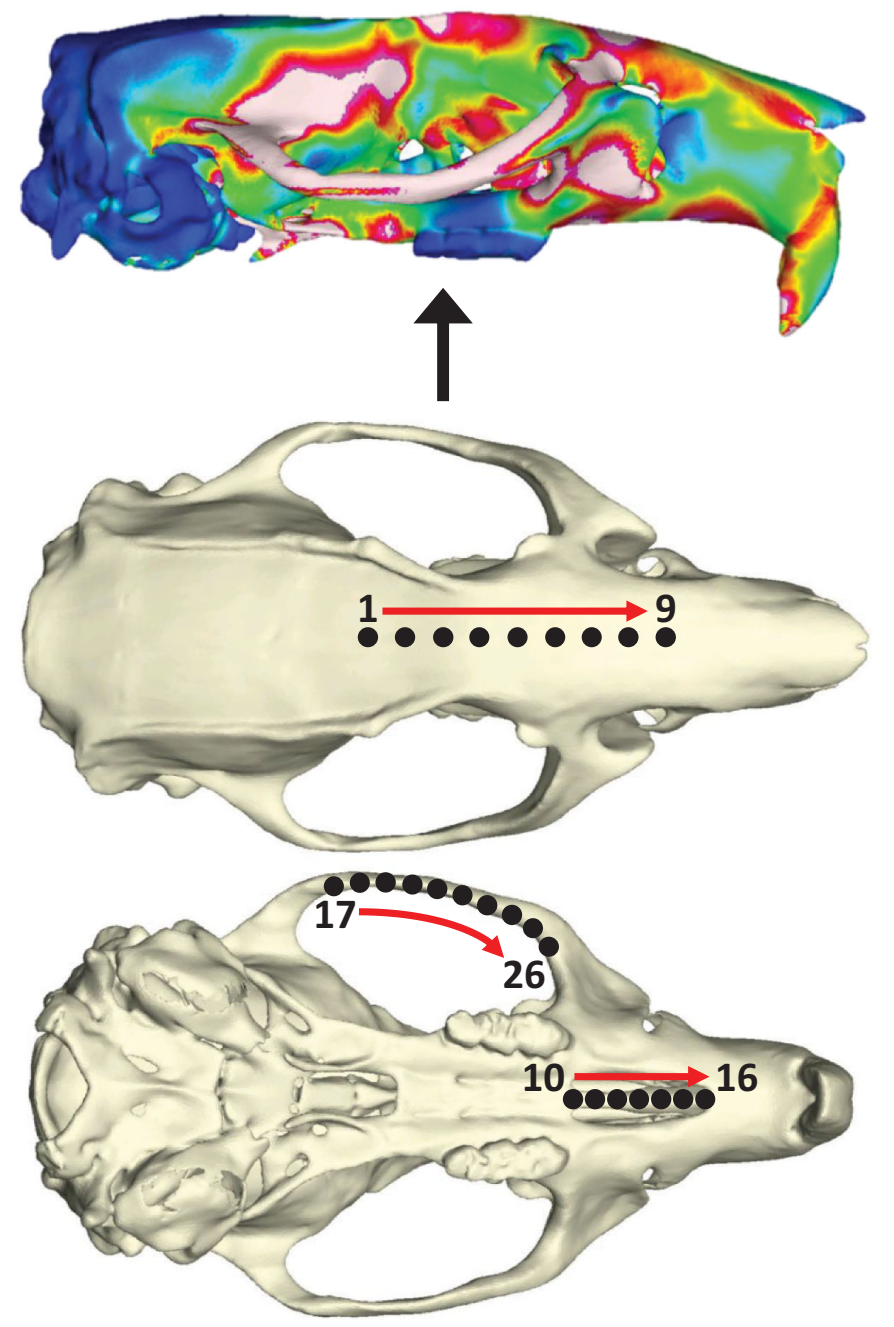
Von Mises stress magnitudes extracted from a total of 26 equidistant landmarks collected from three curves: the anterior neurocranium/muzzle base (1–9), the palate between the anterior palatine foramina (10–16), and the inferior aspect of the right zygomatic arch (17–26).

We then extracted stress magnitudes from equidistant landmarks placed along three curves of the solved models (Fig. 2): (1) nine landmarks from the anterior neurocranium, at the midpoint between the fronto-squamosal intersections at the temporal crests, to the muzzle base at the midpoint between the anterior infraorbital fissures (Richtsmeier et al. 2000), (2) along the palate between the two anterior palatine foramina, and (3) along the inferior right zygomatic arch from the lateral mandibular fossa to the posterior zygomatic root. We assessed the influence of bone remodeling throughout ontogeny on biomechanical performance via von Mises stress, as we are interested in the magnitude of forces dispersed throughout the adult crania of each rat after 16 weeks of feeding. Lower stress magnitudes at a specific location in one group of models relative to another group would indicate a higher volume of bone at that location. Each stress value was the average of the five elements surrounding each landmarked node.

To compare group differences in stress distributed along these semi-landmark curves, permutational multivariate analyses of variance (perMANOVAs) were carried out (1000 permutations) to account for nonuniform variance and nonindependence between semi-landmark sets (Mitchell et al. 2020) using the “adonis2” function in the Vegan R package (v. 2.5.5) (Oksanen et al. 2020). We then carried out pairwise comparisons between groups using the “pairwise.adonis” function from the pairwiseAdonis package (Martinez Arbizu 2020). This test performs a Bonferroni correction on the *p*-values to address the risk of Type 1 errors in multiple tests. Mann–Whitney pairwise U-tests were then performed for all groups at each individual landmark with relaxed Bonferroni-adjusted *p*-values (α = 0.017 or 0.05/3 pairwise comparisons per cohort) (Milne and O'Higgins 2002). Importantly, these final tests were only used as guides to indicate where group differences identified in the initial pairwise tests lie along each curve, since individual tests at each landmark do not account for nonindependence. To visually represent group differences, we presented group mean stress values at all landmarks for each curve as histograms with standard deviation error bars.

## Results

PerMANOVAs were performed on the stress data obtained from each curve of semi-landmarks (Table 2). Stress experienced in the models along each curve was significantly influenced by diet along the muzzle base (*R*^2^ = 0.177, *p* = 0.003), palate (*R*^2^ = 0.205, *p* = 0.001), and ventral zygomatic arch (*R*^2^ = 0.207, *p* = 0.003). The pairwise comparisons indicate significant differences in estimated performance between hard and soft diets along the muzzle base (*R*^2^ = 0.226, *p* = 0.006) and palate (*R*^2^ = 0.221, *p* = 0.006), between the soft and soft:hard diets along the muzzle base (*R*^2^ = 0.180, *p* = 0.042) and palate (*R*^2^ = 0.140, *p* = 0.024), and between the hard and hard:soft diets along the zygomatic arch (*R*^2^ = 0.252, *p* = 0.012) (Table 2).

**Table 2:** PerMANOVA results for mean stress magnitudes and diet for the entire sample, obtained from each landmark along the curves, followed by group pairwise comparisons (significance results [α < 0.05] in bold)

PerMANOVA	*R* ^2^	*F*	*p*
Muzzle	0.177	2.581	**0.003**
Palate	0.205	3.094	**0.001**
Zygomatic arch	0.207	3.133	**0.003**
PerMANOVA pairwise comparisons (adjusted *p*-values).			
^*^ *R* ^2^ effect sizes in brackets.			
	Hard:soft	Soft	Soft:hard
Muzzle			
Hard	1.000 (0.075)	**0.006 (0.226)**	1.000 (0.047)
Hard:soft		0.354 (0.122)	1.000 (0.041)
Soft			**0.042 (0.180)**
Palate			
Hard	0.180 (0.129)	**0.006 (0.221)**	0.204 (0.119)
Hard:soft		0.132 (0.127)	1.000 (0.043)
Soft			**0.024 (0.140)**
Zygomatic arch			
Hard	**0.012 (0.252)**	0.132 (0.157)	1.000 (0.067)
Hard:soft		0.180 (0.149)	0.078 (0.176)
Soft			1.000 (0.064)

**Fig. 3:**
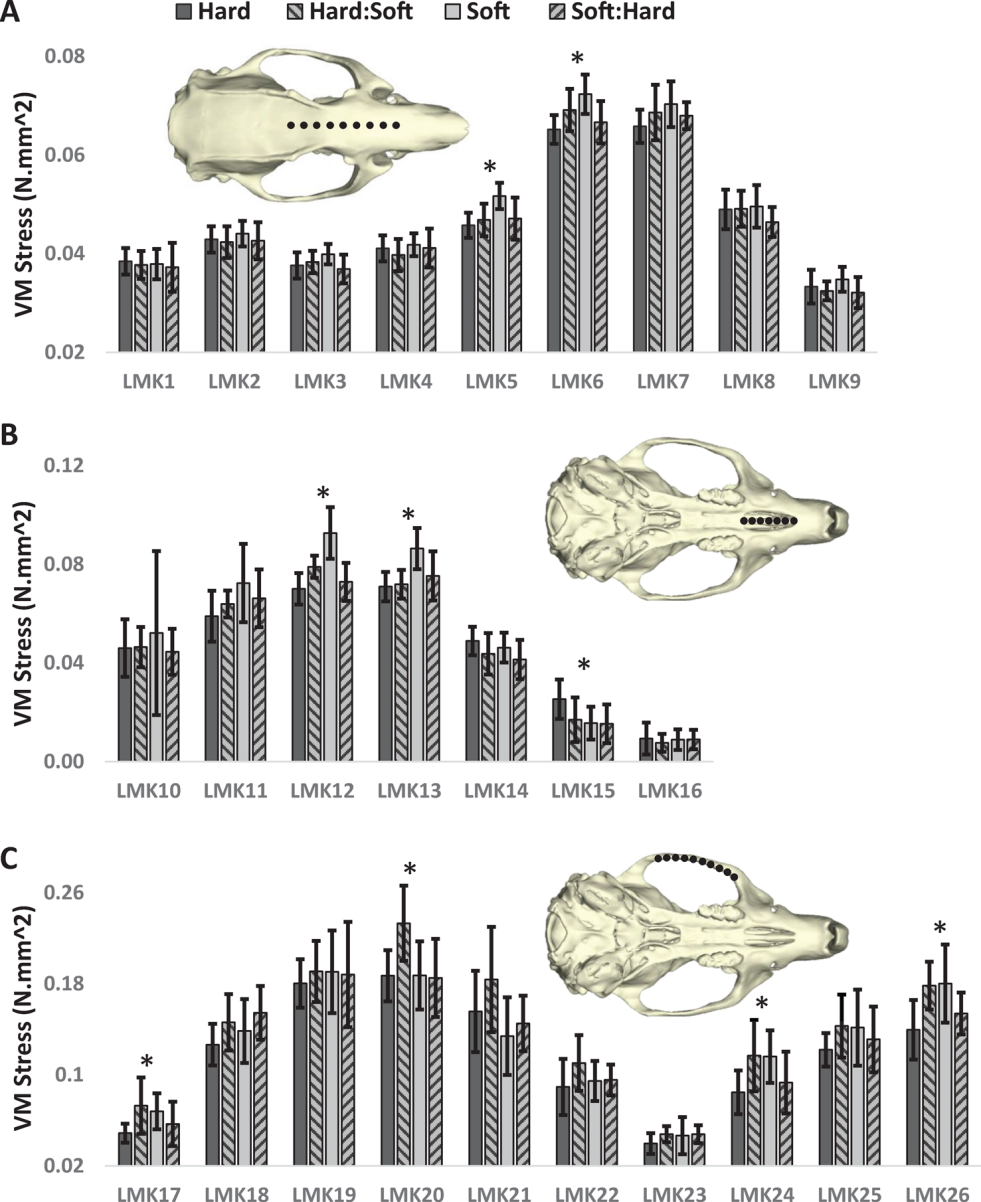
Mean stress magnitudes during an incisor bite at every landmark for each group (*n* = 10). Here, we use “hard” to refer to more mechanically challenging pellets, and “soft” to refer to the ground meal, which requires minimal oral processing. **(A)** Anterior neurocranium/muzzle base, **(B)** palate between anterior palatine foramina, and **(C)** inferior zygomatic arch. Confidence intervals represent standard deviations. Asterisks (*) indicate regions of significant group differences identified in Table 2.

Group mean stress magnitudes and standard deviations for each landmark (Fig. 3) demonstrate consistently similar stress distributions along each curve for all diet groups. However, there are clear differences identifiable via the confidence intervals. As mentioned previously, independent pairwise comparisons for each landmark were not used as a statistical basis for our findings but only as guides to identify the locations of significant differences identified in Table 2. These landmarks are labeled with an asterisk (*). Results for all independent pairwise comparisons are available in the Supporting Information (Table S2).

The curve of the muzzle (Fig. 3A) shows a steady increase in stress from the anterior neurocranium to a peak at the approximate midpoint between the anterior limits of the orbits. The significant differences between hard and soft groups (Table 2) were observed at this peak region of the muzzle base. Greater stress magnitudes are observable for the soft group, specifically at landmarks 5 and 6, which indicate a weaker skull in this region near the rostral frontal bone. The soft group also had significantly higher stress than the soft:hard group at landmark 6. For these two influential landmarks, the group means of the two switched diets were distributed between the means of the hard and soft groups.

The palate (Fig. 3B) demonstrated a general trend across all diet groups of moderate stress at the posterior (sometimes higher due to fine fenestration of the thin palate bone) to a peak near the midpoint, before decreasing to very low magnitudes at the anterior. The significant differences found between hard and soft groups (Table 2) are identifiable at landmarks 12, 13, and 15, near to the mid-region of the anterior palatal foramina. Greater magnitudes are found for the soft diet at landmarks 12 and 13, but the hard diet had higher stress at landmark 15 (albeit at very low stress levels). At landmarks 12 and 13, the mean stresses for the groups that switched diets are distributed between the hard and soft group means but are closer to the hard group on this curve.

Stress along the ventral zygomatic arch (Fig. 3C) is also similar for each group. Magnitudes are lower nearer to the temporomandibular joint and increase steadily to a peak approximately in line with the rear of the cheek tooth row. Stress then decreases to a minimum approximately in line with the posterior M2 molar, before increasing again toward the zygomatic root. The significant differences between hard and hard:soft diet groups (Table 2) are observable at landmarks 17, 20, 24, and 26. In general, the hard, soft, and soft:hard groups are fairly consistent on this curve, although the hard group often occupies the lowest extremes. However, the hard:soft group frequently exhibits the maximum stress values and greatest group means along this curve.

Images of every model used in this study, with their von Mises stress distributions, are available in the Supporting Information (Fig. S1).

## Discussion

Because of the dynamic nature of bone, less mechanically challenging foods offered in captivity may impact the successful reintroduction of captive-reared animals. Here, we tested the influence of food material properties on biomechanical performance among the crania of a single generation of adult rats raised from weaning on different diet regimes. The aim was to determine whether differential loading associated with diets of contrasting textures impacts the development and structural integrity of adult crania. We identify significantly different stress magnitudes within a single population, which are directly associated with dietary variation throughout ontogeny. Our hypothesis that cranial models from rats raised on a less mechanically challenging diet would exhibit significantly greater stresses, during incisor bite simulations than rats fed a more resistant diet was supported at the base of the muzzle and along the palate. However, our results differed somewhat along the inferior zygomatic arch. Instead, along this region, we found that the models of the group that switched as juveniles from hard pellets to soft pellet meal exhibited significantly greater stress than those of the group fed only hard food and recorded the highest mean and absolute stress values along much of the arch. All results highlight the importance of more strenuous feeding behavior in the development of cranial bone mass during ontogeny.

Our results for the muzzle base and palate agree with our predictions based on known mechanisms of bone growth, in that more mechanically challenging diets induced greater bone formation, which resulted in models exhibiting less stress during simulated incisal biting (Fig. 1). In general, bone is deposited under both increased peak strain (from hard biting) and cyclical strain (from chewing), and resorbed under opposite conditions (Frost 1987; Hylander and Johnson 1997). Bone has a site-specific optimal strain environment that is maintained via regular loading activities (Lanyon and Rubin 1985; Biewener 1993; Hylander and Johnson 1997; Ruff et al. 2006; Ravosa et al. 2010, 2016). Elevated strains in the feeding apparatus and limbs stimulate osteogenesis, resulting in either increased bone volume and/or increased bone mineral content (biomineralization). This additional bone formation reduces the amount of strain experienced during a given action or behavior, returning it to within the optimal strain environment. Conversely, reduced loading can facilitate resorption, which will increase the amount of strain, with complete disuse resulting in further bone loss toward a genetically determined minimum bone mass (Skerry 2008). This explains why the landmarks of greatest group differences were located in the regions of greatest stress along the curves of the muzzle base and palate. However, our findings for the zygomatic arch were an unexpected deviation from our hypothesis and suggest that sudden unloading (or disuse) may, in certain regions of the skull, be detrimental to development in some animals.

Some of the most relevant research on unloading are studies of the effects of spaceflight and simulated antigravity scenarios on skeletal bone. Sudden and prolonged unloading of bone has been shown to lead to decreases in the number and activity of osteoblasts responsible for bone deposition (Bikle and Halloran 1999; Nagaraja and Risin 2013). Under such conditions, mesenchymal stem cells are instead more likely to differentiate into adipocytes rather than osteoblasts (Keune et al. 2016), which will affect rates of subsequent bone formation. In some instances, this reduction in osteoblast activity may adversely affect the relative robusticity of immature, developing bone if suddenly unloaded for an extended period during ontogeny. This is particularly relevant to the care of juvenile wild animals rescued from illness or injury. Our results suggest that a switch from a wild, variable diet to less challenging foods provisioned through to adulthood can impair growth and development in some regions of the skull, which may inhibit skeletal integrity and performance upon reintroduction. Such divergent cranial phenotypes will likewise respond differently to natural selection, which will influence evolutionary patterns of morphological change in subsequent generations (Ravosa et al. 2016).

The close association between the zygomatic arch and the masseter muscle complex might explain why sudden, prolonged unloading affected this region more than at the muzzle base and palate (Yamada and Kimmel 1991). Muscle atrophy precedes bone resorption upon unloading. The deep masseter origin runs the length of the inferior zygomatic arch and any muscle atrophy induced by unloading may exacerbate the development of skeletal deficits (Lloyd et al. 2014). Yamada and Kimmel (1991) observed a similar relationship between muscle function and bone formation along the mandibular ramus of rats, in which a mild decrease in muscle use among growing rats fed a softer diet led to underdevelopment of the periosteal surface around the muscle insertions. As no muscle origins are located at the muzzle base or palate, we suggest these regions are at less risk of this effect. Furthermore, should a reintroduced animal that has experienced sudden unloading during ontogeny attempt to immediately utilize more resistant resources in the wild, the faster recovery rate of muscle compared with bone presents a risk of skeletal injury to the weakened zygomatic arch (Allen et al. 2006). This, coupled with the greater observed instances of oral pathologies in captive animals associated with less mechanically challenging loading regimes (Corruccini and Beecher 1982; Fitch and Fagan 1982; Ciochon et al. 1997; Crossley and del Mar Miguélez 2001; O'Regan and Kitchener 2005), suggests a more difficult path for animals raised on diets that are overly dependent on less mechanically challenging, processed foods during rearing and may hinder the ability of a captive-reared animal to effectively transition to wild foods upon release. Our results therefore suggest that a more variable, challenging diet resulting in greater peak strains and/or more cyclical strains throughout all stages of ontogeny can mitigate such unfavorable outcomes.

Whether the observed differences in biomechanical performance between groups are retained throughout adult life or are reversible cannot be determined from this study. The differences we have identified are almost certainly associated with both modeling (development) and remodeling (Haversian mechanisms) (Turner et al. 1995; Pearson and Lieberman 2004), but the relative contribution remains to be determined (Lad et al. 2021). Given that modeling during the juvenile growth period is largely responsible for the addition of bone mass, this mechanism likely contributes to the variation in bone volume and thus stress differences exhibited by our finite element models. By adulthood, bone formation via modeling ceases and remodeling is differentially more important (Bertram and Swartz 1991; Turner et al. 1995). Gelbke (1951) stated that changes to bone morphology appear to be largely reversible if influential forces are removed prior to skeletal maturation. At least for the muzzle and palate, we note here that the groups with switched diets evidenced bone morphology more reflective of the new diet. In addition, it has been demonstrated that older individuals can form as much bone as younger individuals, albeit through a slower process and differentially at certain skeletal sites (Turner et al. 1995; Scott et al. 2014). However, it is well established that cortical bone is more responsive to strain prior to skeletal maturity (Pearson and Lieberman 2004; Ravosa et al. 2016), with Donahue et al. (2001) noting that osteoblasts in older rats are less sensitive than younger individuals to mechanical signals from flow-induced calcium ion oscillations. To what extent biomechanical performance converges on that of the respective diet through adulthood cannot be determined without also modeling older individuals. Regardless, the rats studied here represent a fully mature stage of development (Roach et al. 2003) and the bones of adult rodents have been shown to be still not fully recovered from the effects of unloading after 12 weeks (Grimston et al. 2007). Therefore, if the animals studied here were to be released into the wild, the soft diet group would likely be at a disadvantage compared with the hard diet group, as their mechanically weaker crania would, at least temporarily, limit access to some more resistant foods that the hard diet group could immediately exploit. Any reversible effects may outlast less productive seasons and increase the chances of mortality when desirable softer, nutrient-rich foods are rare.

It is possible that, over time, bone mineral density, or biomineralization, could also play a role in the recovery of resorbed or malformed bone in released individuals. Our simulations here were focused solely on bone architecture (quantity) and did not factor in variation in bone quality. Yet, biomineralization is known to occur with variation in dietary composition and masticatory loading (Ravosa et al. 2008a, 2008b; Kohn et al. 2009; Franks et al. 2016, 2017). The result of elevated mineral content in bone is increased stiffness, which results in decreased strain (or deformation) during loading (Lanyon and Rubin 1985; Currey 2003). Therefore, both bone architecture and bone material properties may impact the outcomes of performance simulations. Given that biomineralization is encouraged under similar conditions to bone deposition, it is possible that if our models incorporated both bone architecture and bone mineralization properties, this would serve to more clearly define group differences shown by our current models. However, in instances where no significant differences are found in macrostructure (e.g., bone architecture) between intraspecific groups with different diets, the existence of microstructural changes to bone quality should not be discounted (e.g., Franks et al. 2017). For example, previous dietary plasticity work in the rabbit zygomatic arch has found both changes in cross-sectional shape (Menegaz et al. 2010) and tissue mineral density (Franks et al. 2016) but not an increase in bone mass via cortical bone deposition. Should future studies focus on the recovery of bone in adults that return to a hard diet, analyses that incorporate bone materials properties and/or histological data could potentially determine the extent to which microstructural variation also plays a role in feeding performance.

## Conclusion

This study demonstrates that diets incorporating more challenging foods are likely beneficial in the rearing of wildlife for reintroduction. Long-term consumption of a diet that reduces strain or cyclical loads is shown here to result in increased stress throughout the skull when biting, compared with individuals raised on more resistant food items. This may limit access to certain resources upon release, especially during less productive seasons. Furthermore, although early bone strength conditioning is certainly beneficial, we show that in some cases, a sudden and prolonged relaxing of loads can be detrimental to bone formation as well. This is of particular relevance to rescued juveniles, suggesting that a switch in captivity to less challenging foods during mid-development could pose a significant risk to success in the wild. These results are important for developing optimized approaches to rearing animals for release that consider not just nutritional needs, but developmental requirements as well. Although we cannot quantify here the extent to which these findings may apply to other species, they are a product of physiological mechanisms common to disparate vertebrate taxa. It is therefore likely a best practice initiative to assume that dietary constituents throughout development will play a role in the structure of the adult skull for most animals that orally process their food items. It is therefore advisable that measures be taken, where possible, to imitate the known dietary ecology of the wildlife in care as much as possible, including food nutrition, texture, and seasonality, in order to condition bones and muscles for tasks expected in the wild. This can reduce some deleterious physiological, anatomical, and performance-related effects imparted by a captive environment and help facilitate the ultimate goal of preparation for reintroduction.

## Supplementary Material

obab030_Supplemental_Figure_Tables

## Data Availability

STLs of all crania and mandibles are available on MorphoSource at https://www.morphosource.org/projects/000385099.
