# Supplementary material for: More Challenging Diets Sustain Feeding Performance: Applications Toward the Captive Rearing of Wildlife
Source: Integr Org Biol. 2021 Nov 22;3(1):obab030. doi: 10.1093/iob/obab030 (PMC8653637; doi:10.1093/iob/obab030)
Supplement: obab030_Supplemental_Figure_Tables [file obab030_supplemental_figure_tables.zip › Figure S1.pdf]

Figure S1: All solved models (dorsal, lateral, ventral views). Heat maps set to a maximum threshold of 0.07N.mm^2 for visual contrasts. G1 = Hard/Pellet diet; G2 = Hard to soft/Pellet to meal diet; G3 = Soft/Meal diet; G4 = Soft to hard/Meal to pellet diet.

G1A1

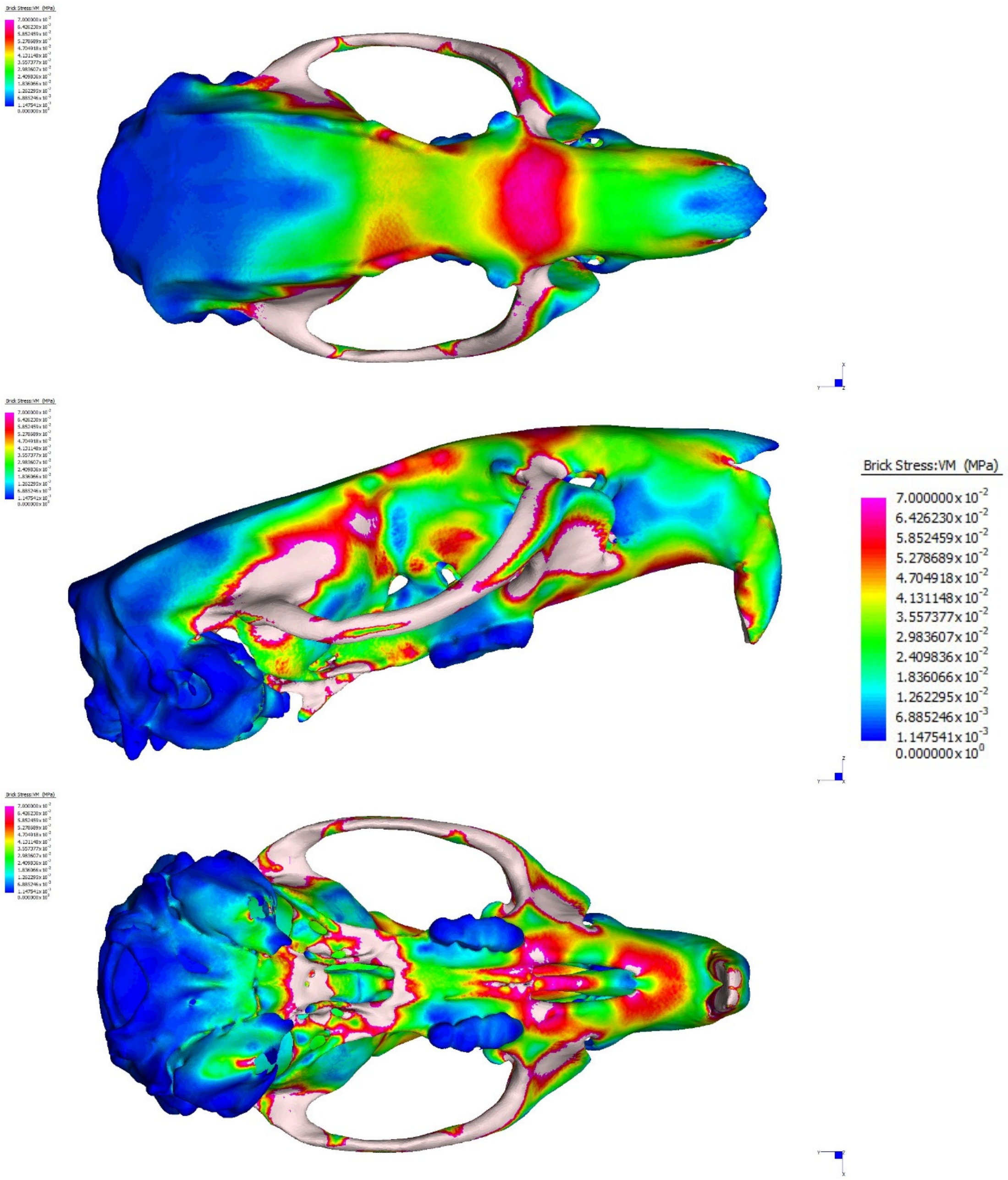

G1A2

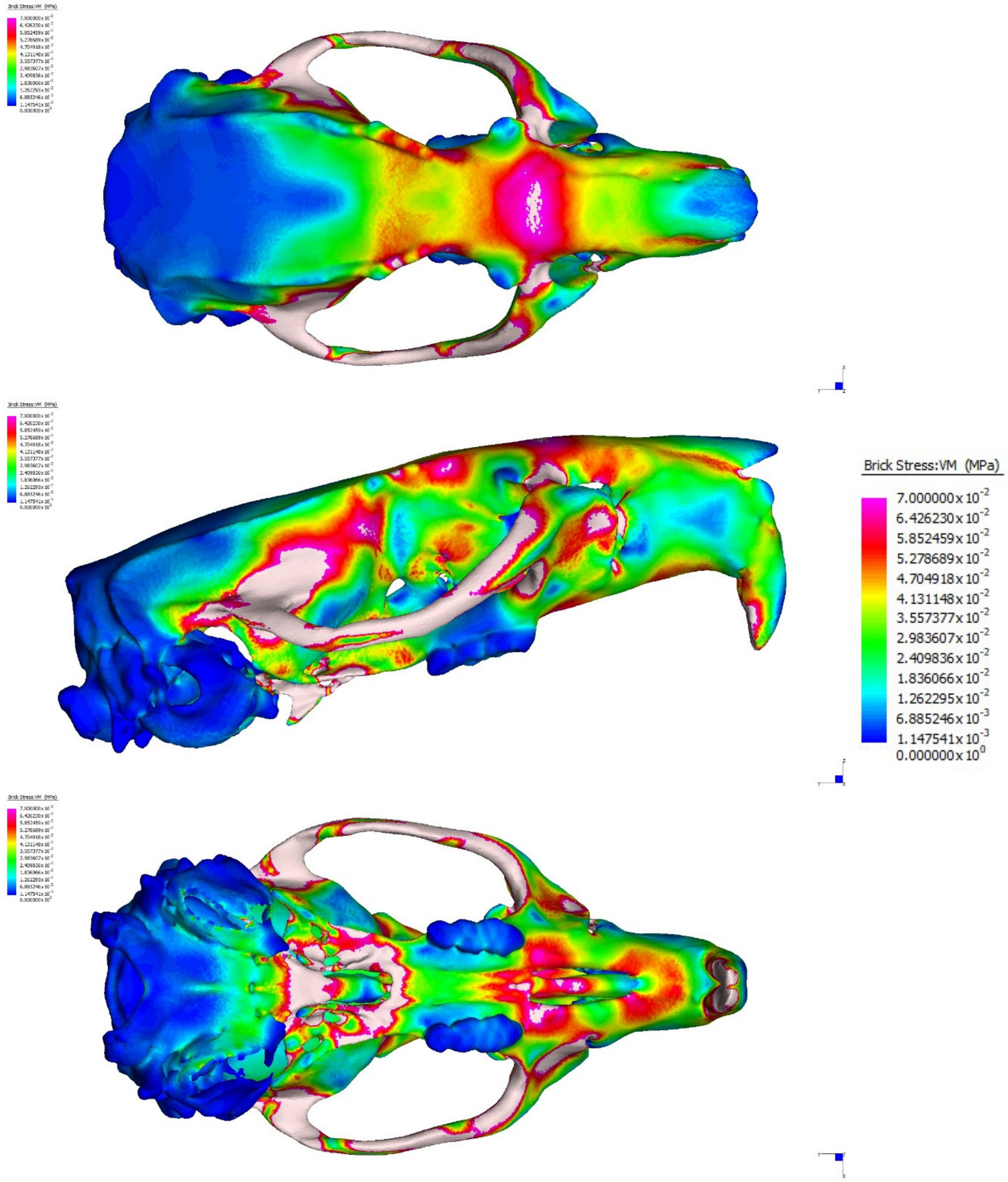

Brick Stress:VM (MPa)
7.000000×10^{-2}
6.426230×10^{-2}
5.852459×10^{-2}
5.278689×10^{-2}
4.704918×10^{-2}
4.131148×10^{-2}
3.557377×10^{-2}
2.983607×10^{-2}
2.409836×10^{-2}
1.836066×10^{-2}
1.262295×10^{-2}
6.885246×10^{-3}
1.147541×10^{-3}
0.000000×10^{0}

G1A3

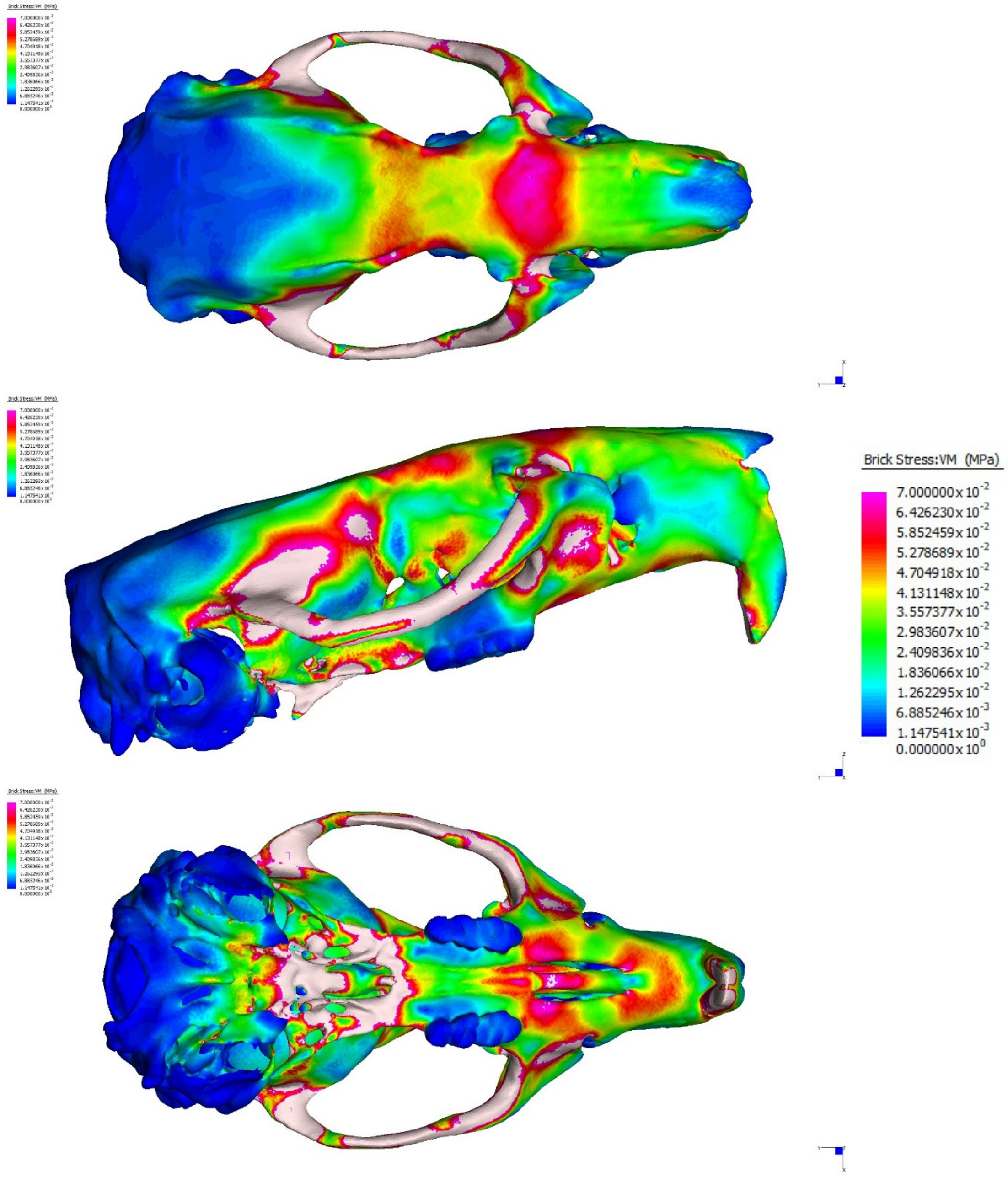
Brick Stress:VM (MPa)
7.000000×10^{-2}
6.426230×10^{-2}
5.852459×10^{-2}
5.278689×10^{-2}
4.704918×10^{-2}
4.131148×10^{-2}
3.557377×10^{-2}
2.983607×10^{-2}
2.409836×10^{-2}
1.836066×10^{-2}
1.262295×10^{-2}
6.885246×10^{-3}
1.147541×10^{-3}
0.000000×10^{0}

G1A4

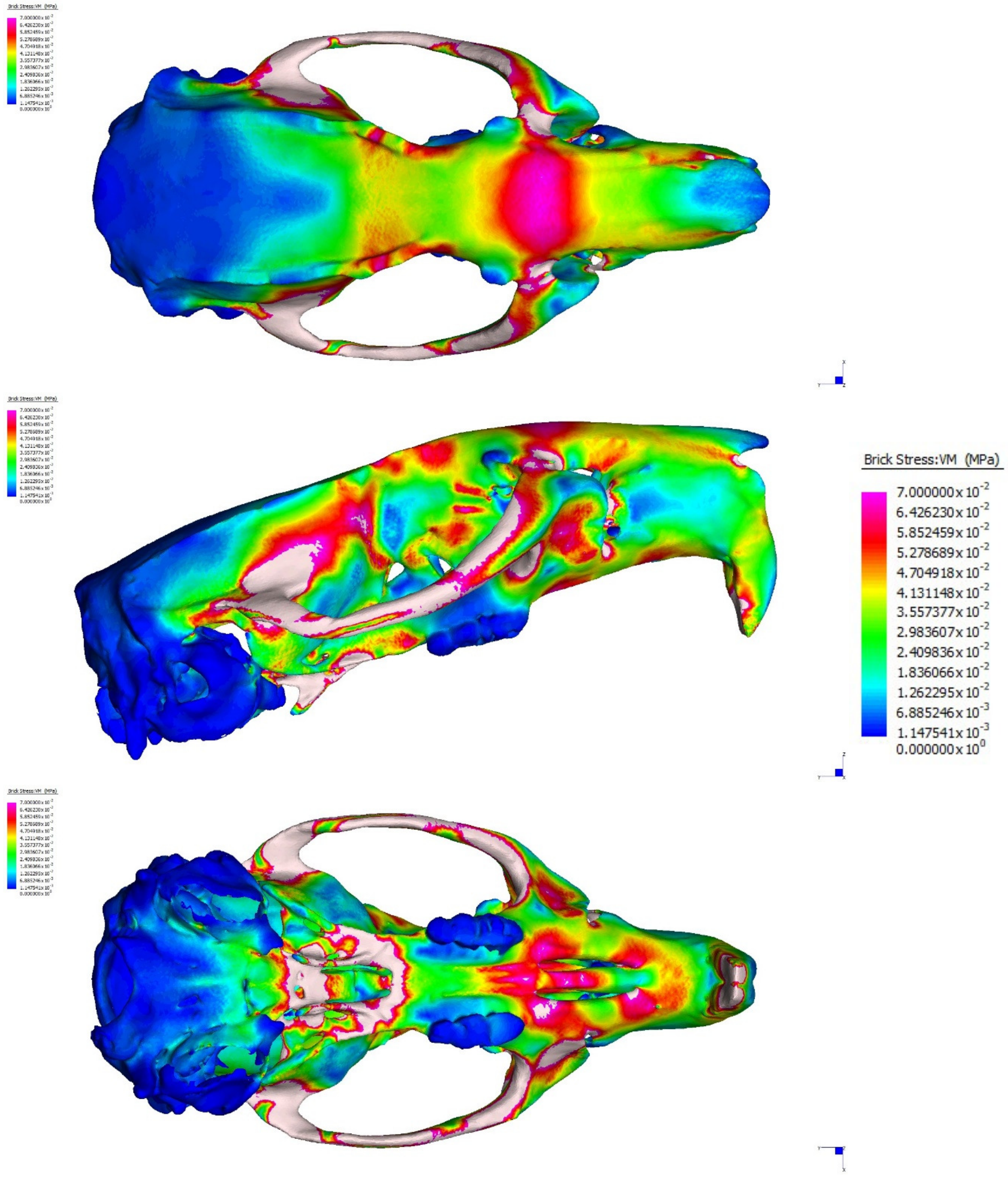
Brick Stress:VM (MPa)
7.000000x10^-2
6.426230x10^-2
5.852459x10^-2
5.278689x10^-2
4.704918x10^-2
4.131148x10^-2
3.557377x10^-2
2.983607x10^-2
2.409836x10^-2
1.836066x10^-2
1.262295x10^-2
6.885246x10^-3
1.147541x10^-3
0.000000x10^0

G1A5

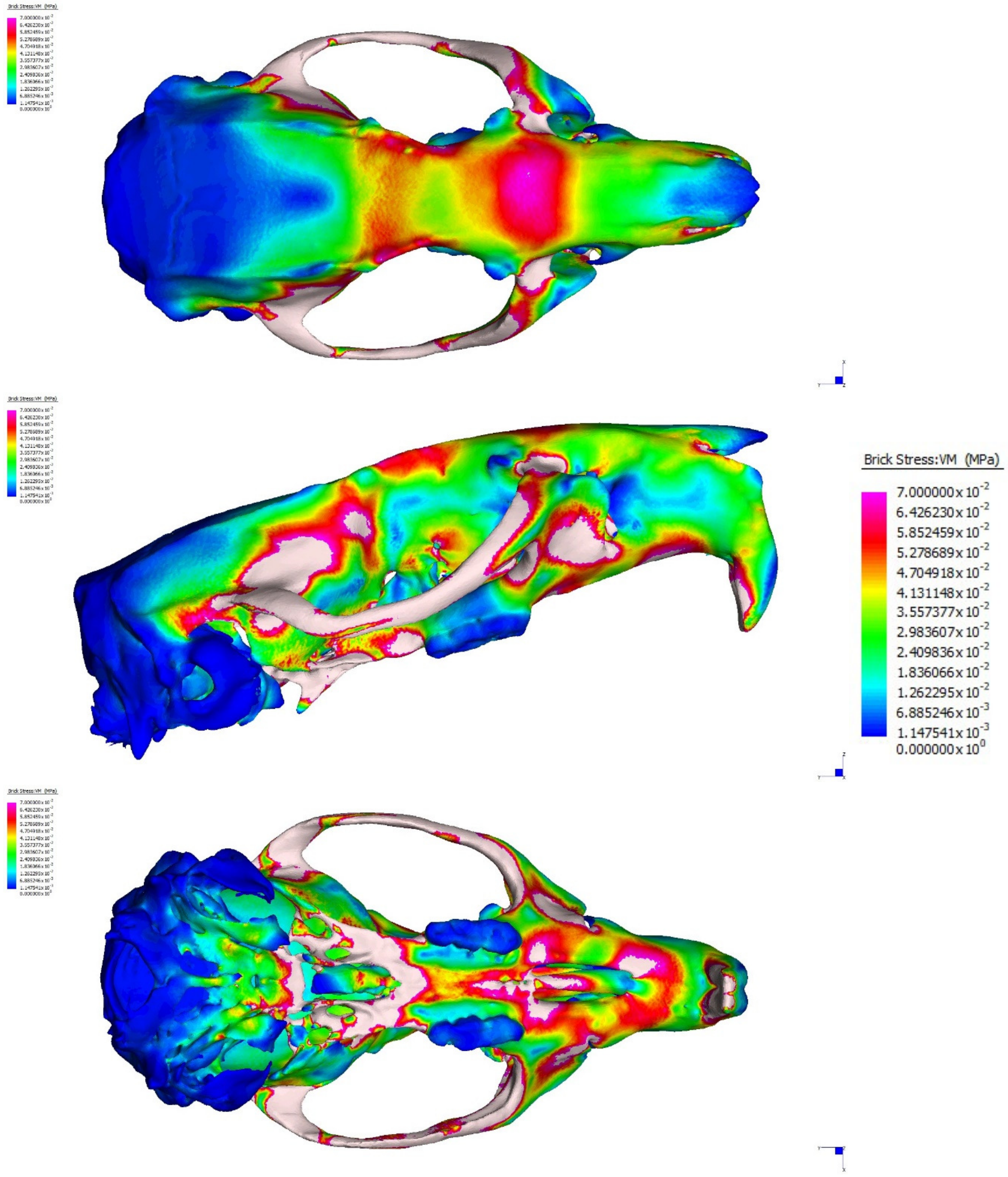

Brick Stress:VM (MPa)
7.000000x10^-2
6.426230x10^-2
5.852459x10^-2
5.278689x10^-2
4.704918x10^-2
4.131148x10^-2
3.557377x10^-2
2.983607x10^-2
2.409836x10^-2
1.836066x10^-2
1.262295x10^-2
6.885246x10^-3
1.147541x10^-3
0.000000x10^0

G1A6

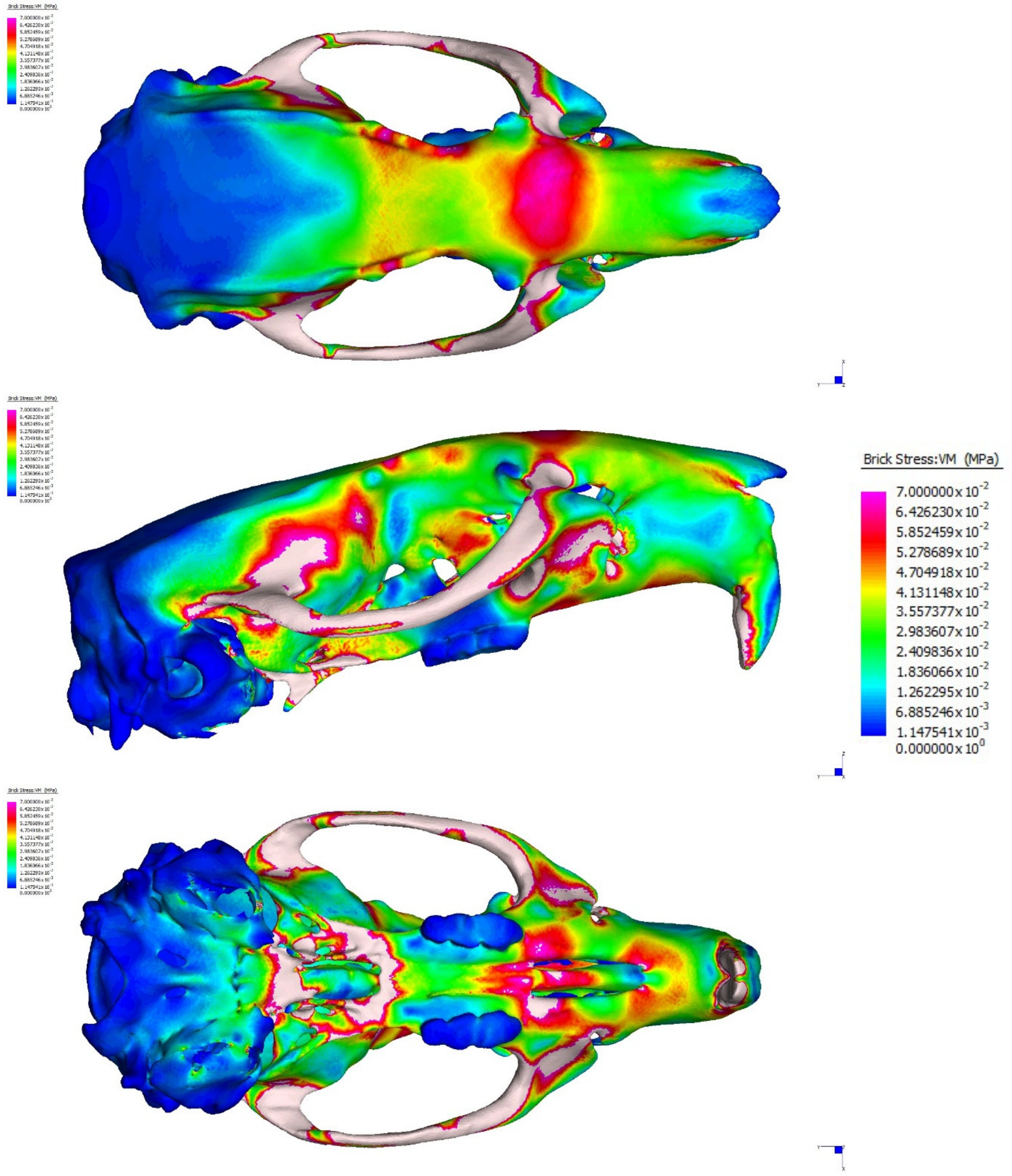
Brick Stress:VM (MPa)
7.000000x10^-2
6.426230x10^-2
5.852459x10^-2
5.278689x10^-2
4.704918x10^-2
4.131148x10^-2
3.557377x10^-2
2.983607x10^-2
2.409836x10^-2
1.836066x10^-2
1.262295x10^-2
6.885246x10^-3
1.147541x10^-3
0.000000x10^0

G1A7

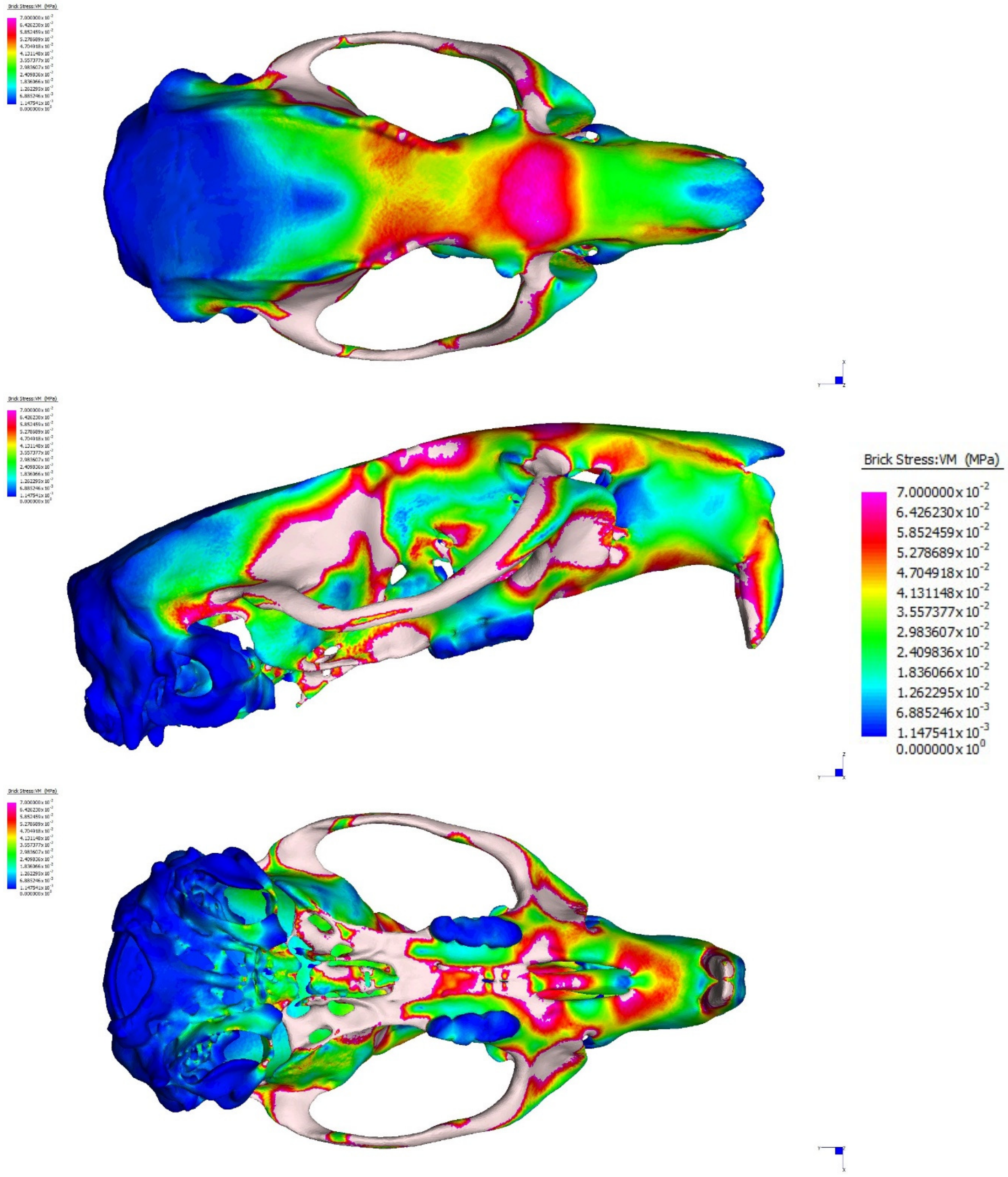
Brick Stress:VM (MPa)
7.000000x10^-2
6.426230x10^-2
5.852459x10^-2
5.278689x10^-2
4.704918x10^-2
4.131148x10^-2
3.557377x10^-2
2.983607x10^-2
2.409836x10^-2
1.836066x10^-2
1.262295x10^-2
6.885246x10^-3
1.147541x10^-3
0.000000x10^0

G1A8

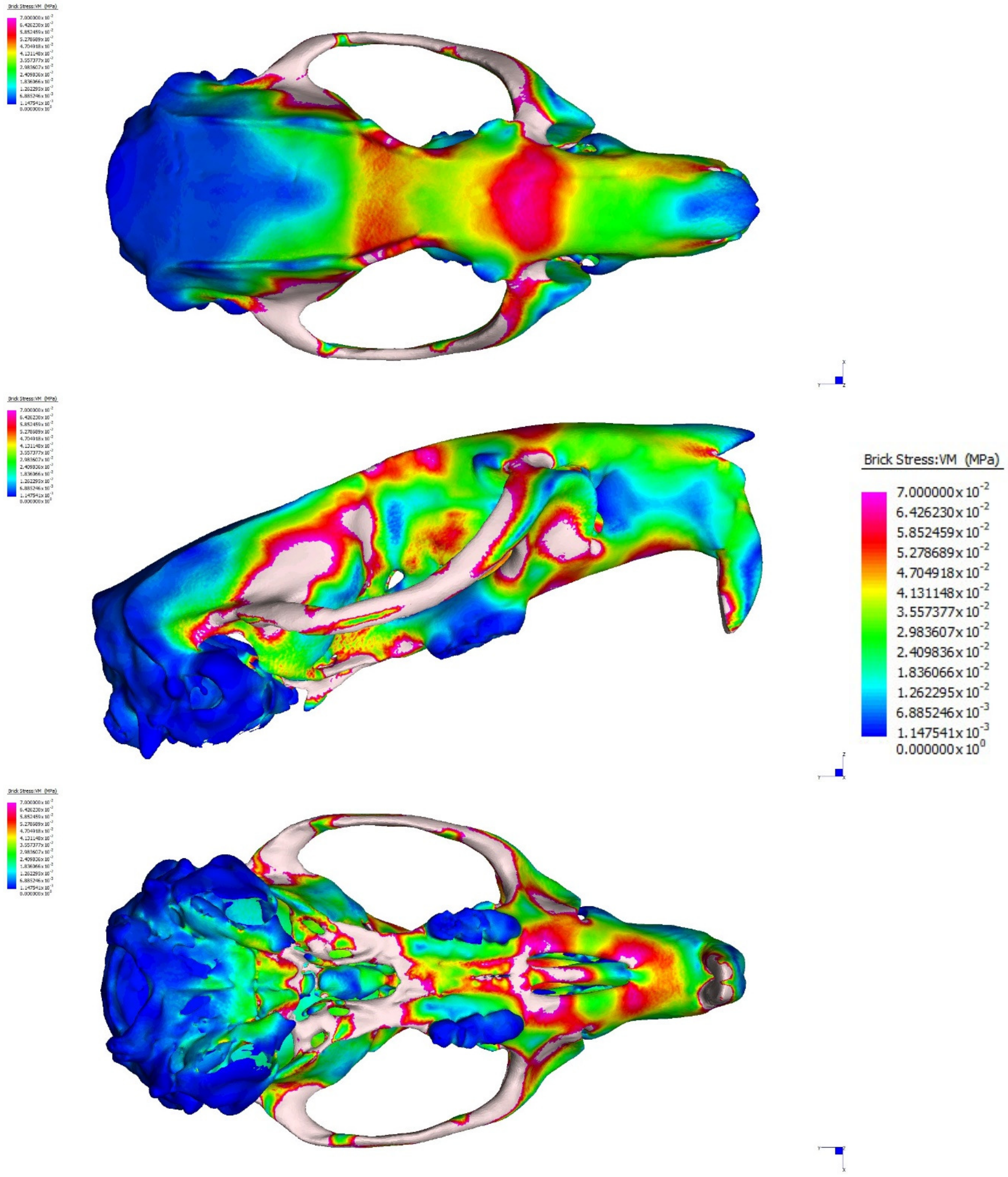
Brick Stress:VM (MPa)
7.000000x10^-2
6.426230x10^-2
5.852459x10^-2
5.278689x10^-2
4.704918x10^-2
4.131148x10^-2
3.557377x10^-2
2.983607x10^-2
2.409836x10^-2
1.836066x10^-2
1.262295x10^-2
6.885246x10^-3
1.147541x10^-3
0.000000x10^0

G1A9

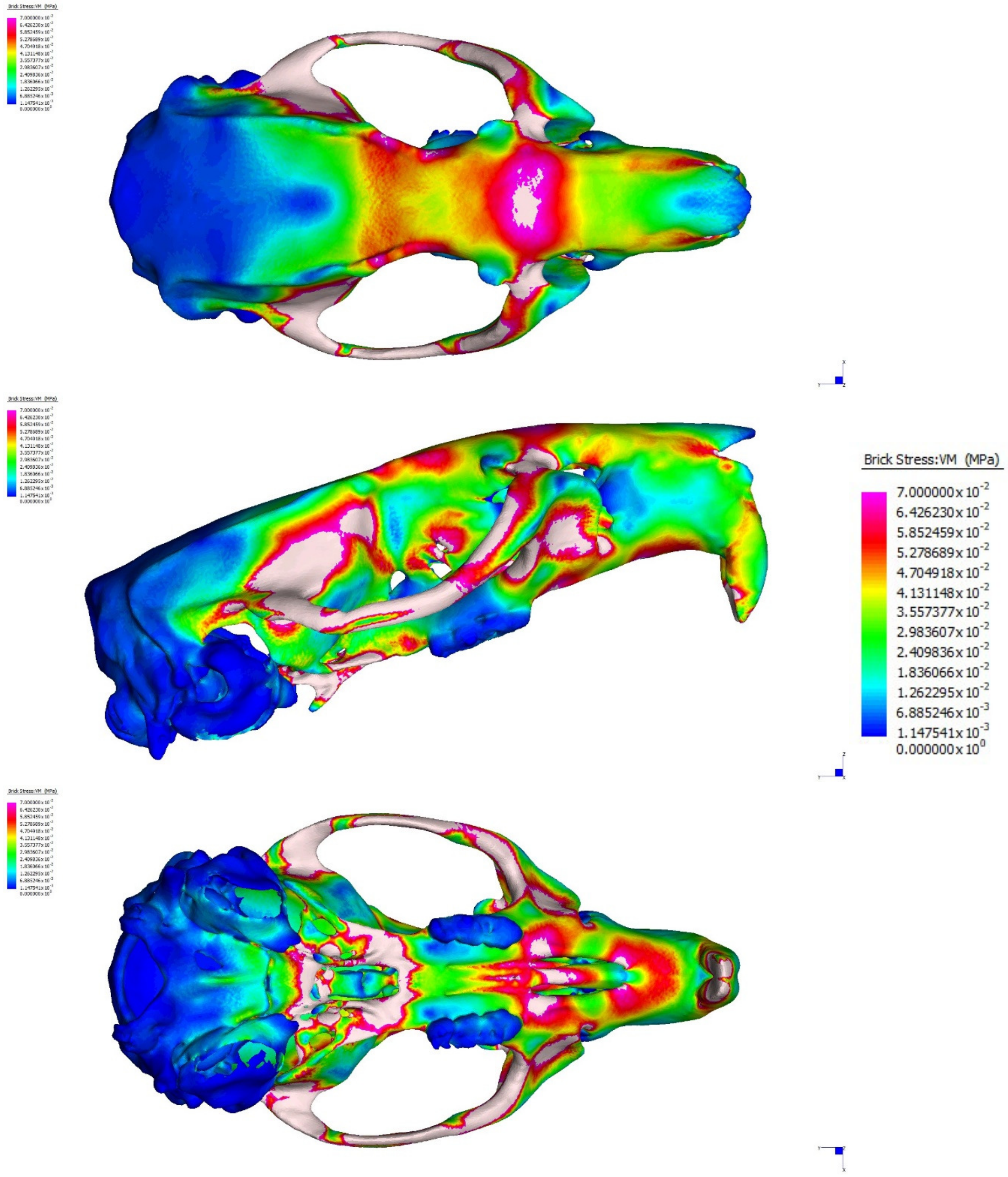
Brick Stress:VM (MPa)
7.000000x10^-2
6.426230x10^-2
5.852459x10^-2
5.278689x10^-2
4.704918x10^-2
4.131148x10^-2
3.557377x10^-2
2.983607x10^-2
2.409836x10^-2
1.836066x10^-2
1.262295x10^-2
6.885246x10^-3
1.147541x10^-3
0.000000x10^0

G1A10

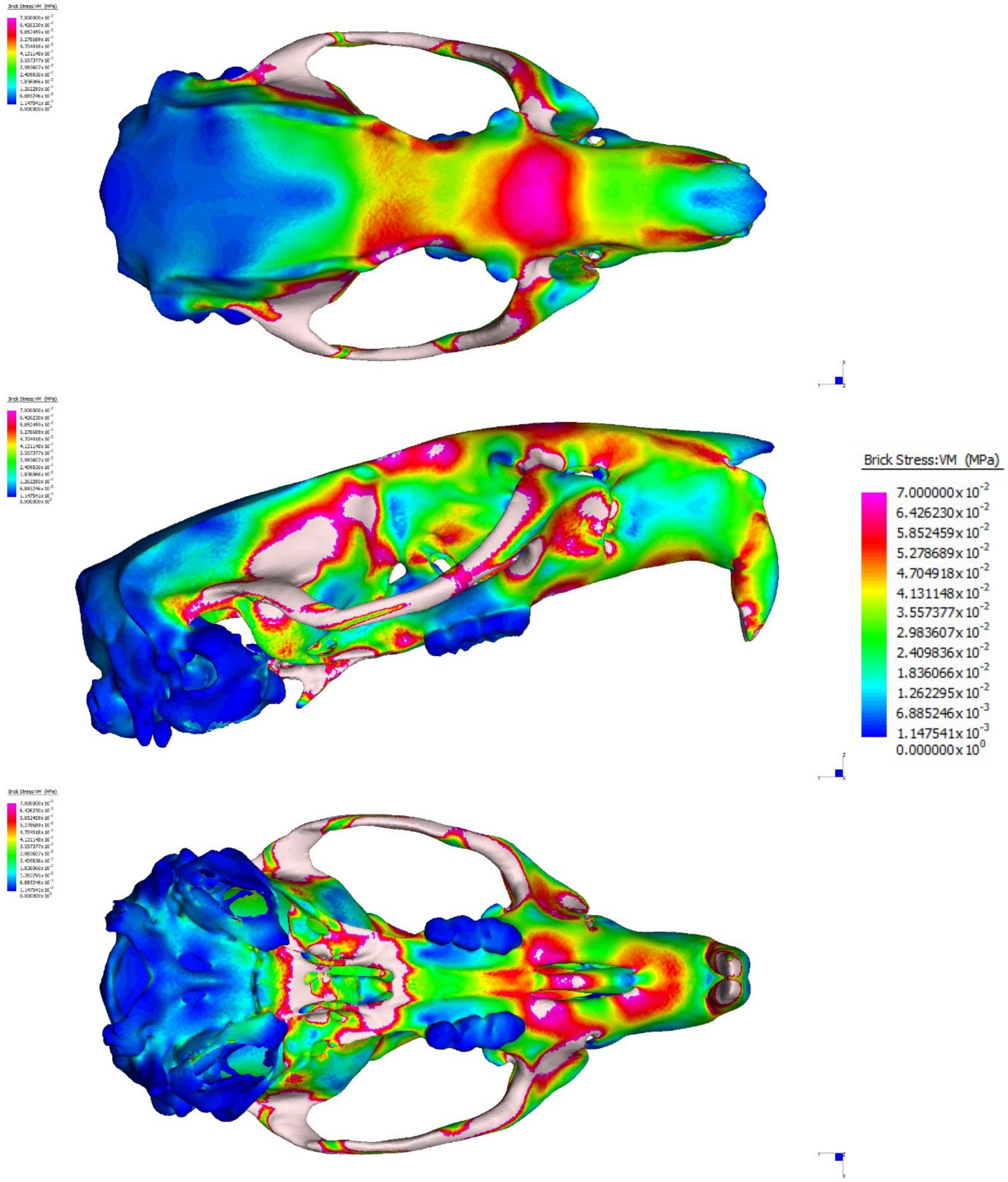

Brick Stress:VM (MPa)
7.000000×10^{-2}
6.426230×10^{-2}
5.852459×10^{-2}
5.278689×10^{-2}
4.704918×10^{-2}
4.131148×10^{-2}
3.557377×10^{-2}
2.983607×10^{-2}
2.409836×10^{-2}
1.836066×10^{-2}
1.262295×10^{-2}
6.885246×10^{-3}
1.147541×10^{-3}
0.000000×10^{0}

G2A1

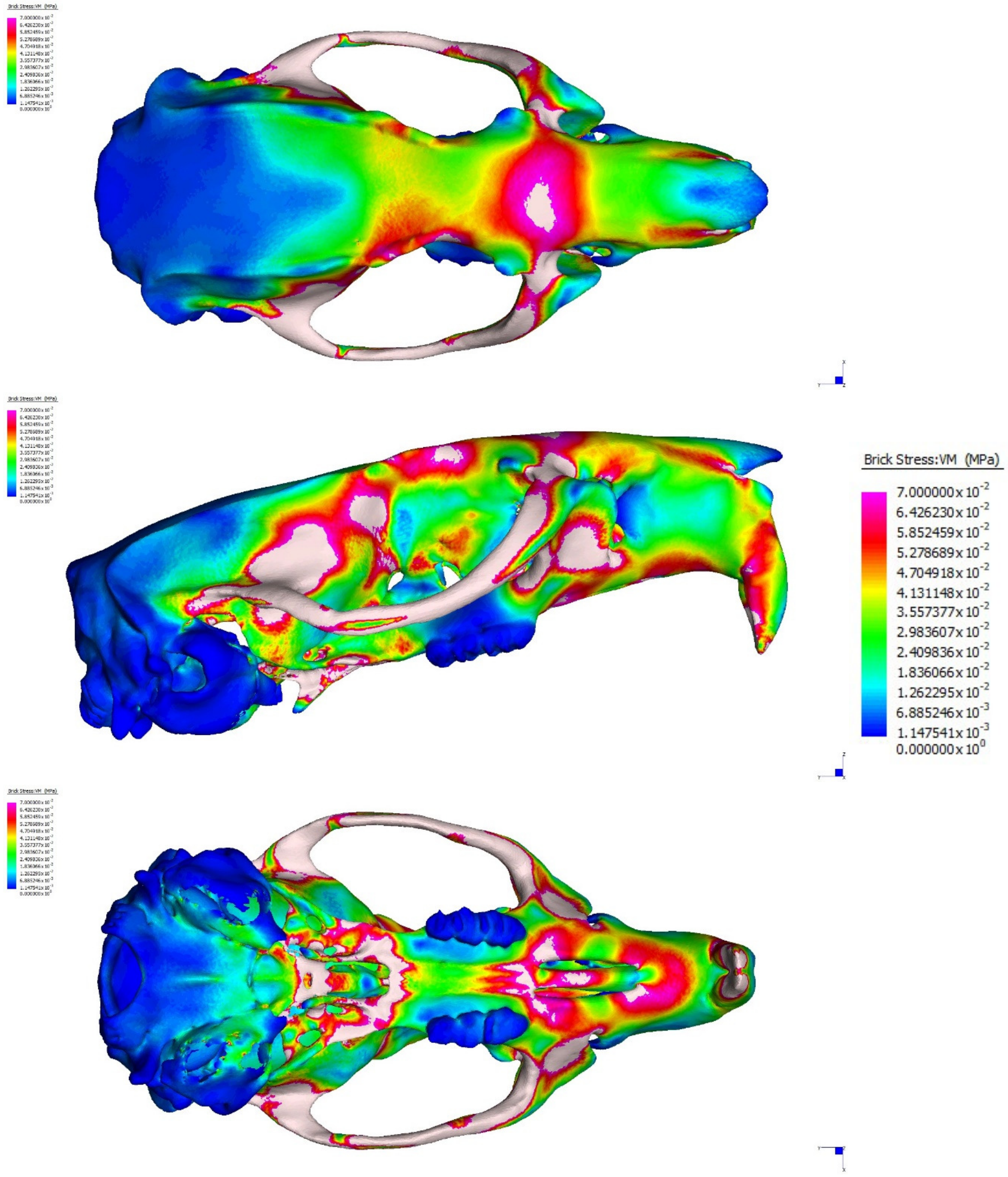

Brick Stress:VM (MPa)
$7.000000 \text{x} 10^{-2}$
$6.426230 \text{x} 10^{-2}$
$5.852459 \text{x} 10^{-2}$
$5.278689 \text{x} 10^{-2}$
$4.704918 \text{x} 10^{-2}$
$4.131148 \text{x} 10^{-2}$
$3.557377 \text{x} 10^{-2}$
$2.983607 \text{x} 10^{-2}$
$2.409836 \text{x} 10^{-2}$
$1.836066 \text{x} 10^{-2}$
$1.262295 \text{x} 10^{-2}$
$6.885246 \text{x} 10^{-3}$
$1.147541 \text{x} 10^{-3}$
$0.000000 \text{x} 10^{0}$

G2A2

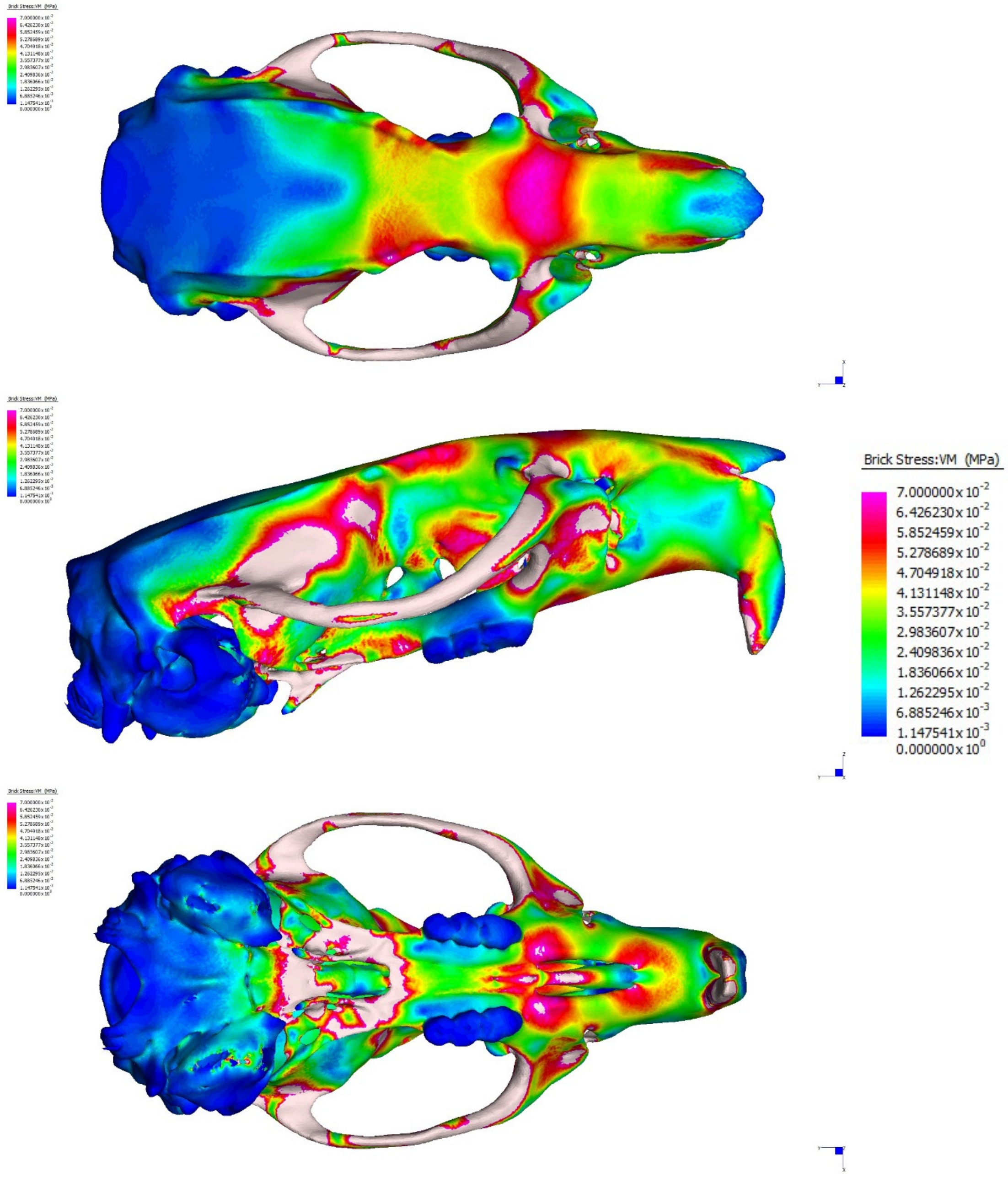
Brick Stress:VM (MPa)
7.000000x10^-2
6.426230x10^-2
5.852459x10^-2
5.278689x10^-2
4.704918x10^-2
4.131148x10^-2
3.557377x10^-2
2.983607x10^-2
2.409836x10^-2
1.836066x10^-2
1.262295x10^-2
6.885246x10^-3
1.147541x10^-3
0.000000x10^0

G2A3

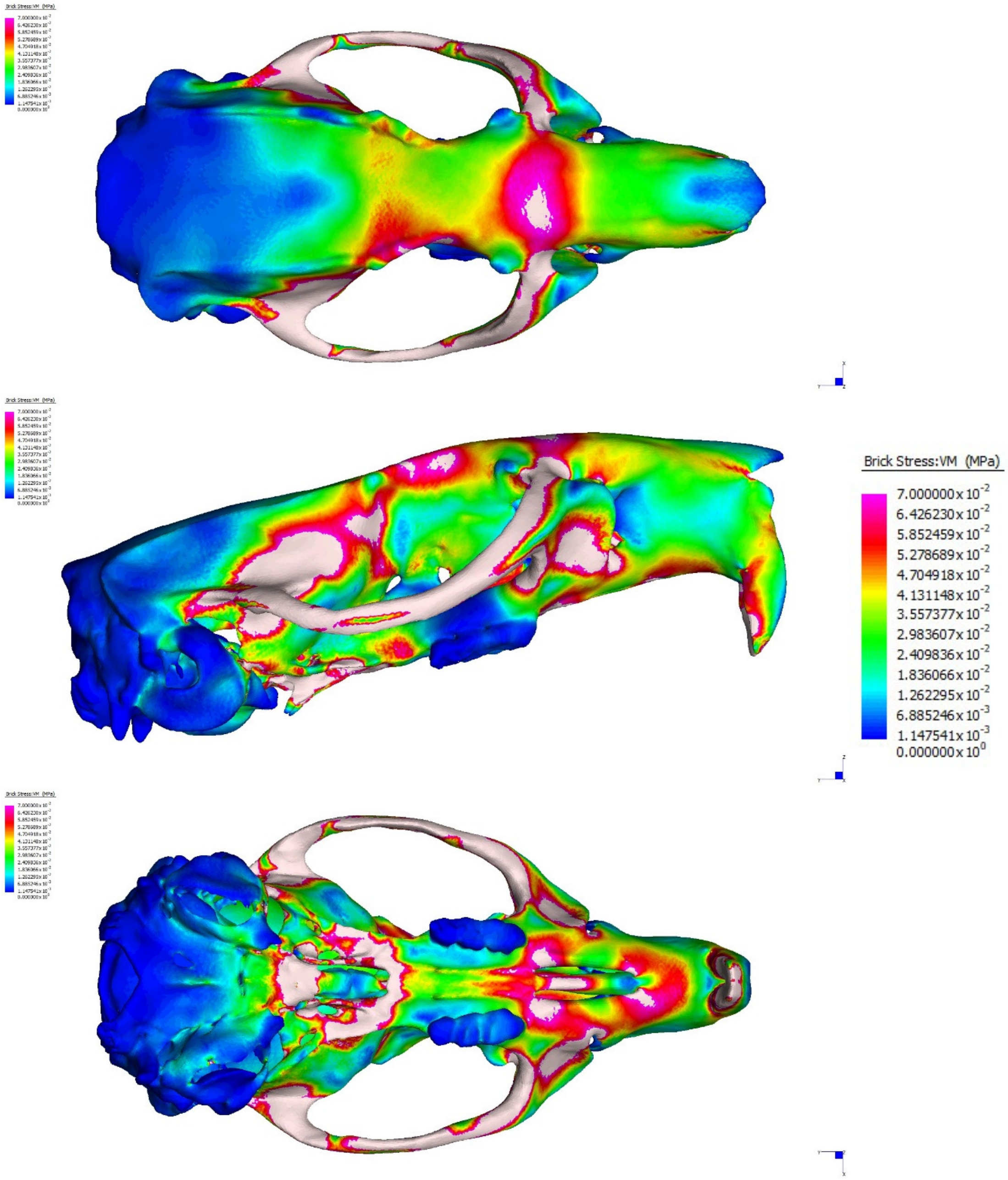
Brick Stress:VM (MPa)
7.000000x10^-2
6.426230x10^-2
5.852459x10^-2
5.278689x10^-2
4.704918x10^-2
4.131148x10^-2
3.557377x10^-2
2.983607x10^-2
2.409836x10^-2
1.836066x10^-2
1.262295x10^-2
6.885246x10^-3
1.147541x10^-3
0.000000x10^0

G2A4

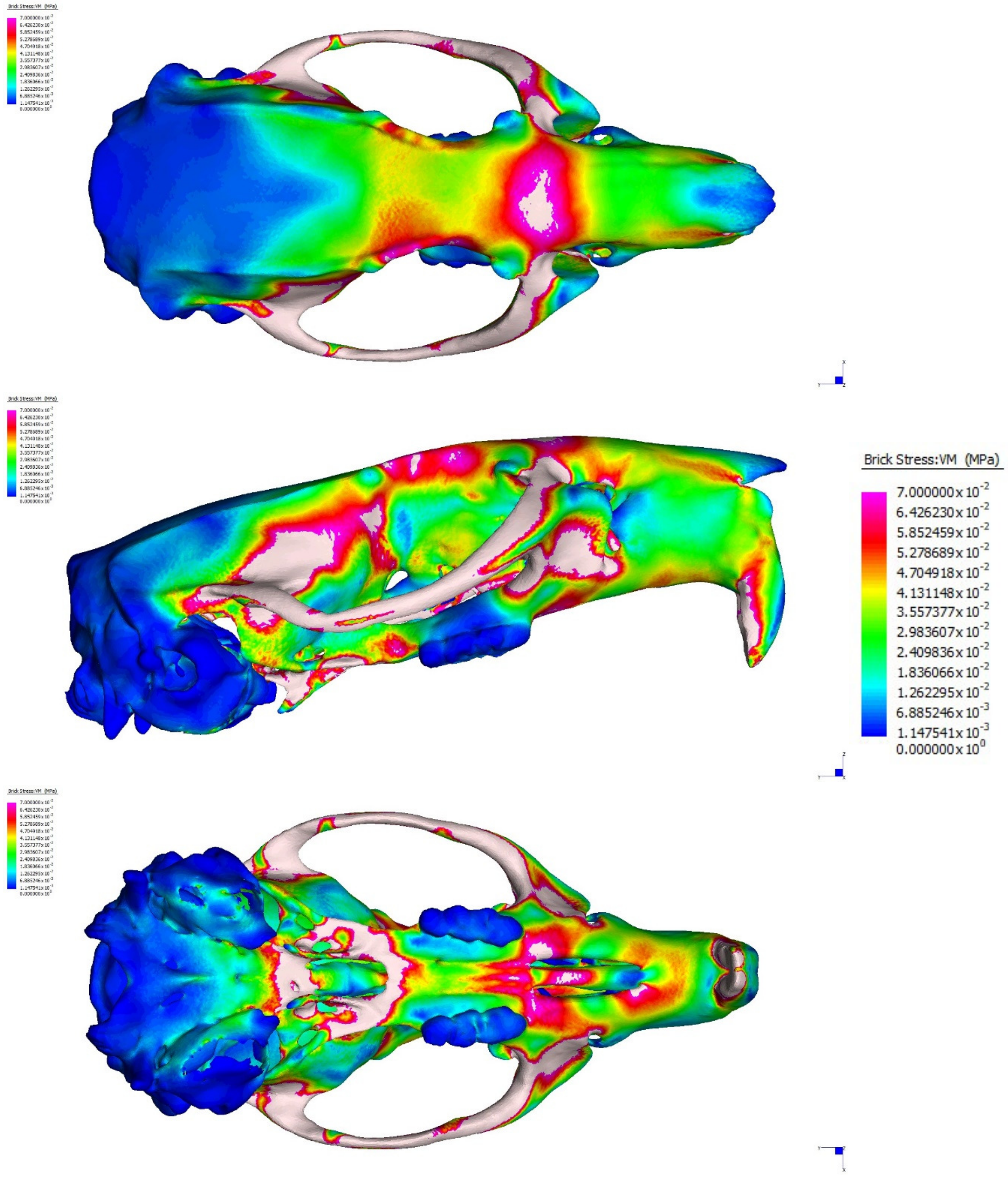
Brick Stress:VM (MPa)
7.000000x10^-2
6.426230x10^-2
5.852459x10^-2
5.278689x10^-2
4.704918x10^-2
4.131148x10^-2
3.557377x10^-2
2.983607x10^-2
2.409836x10^-2
1.836066x10^-2
1.262295x10^-2
6.885246x10^-3
1.147541x10^-3
0.000000x10^0

G2A5

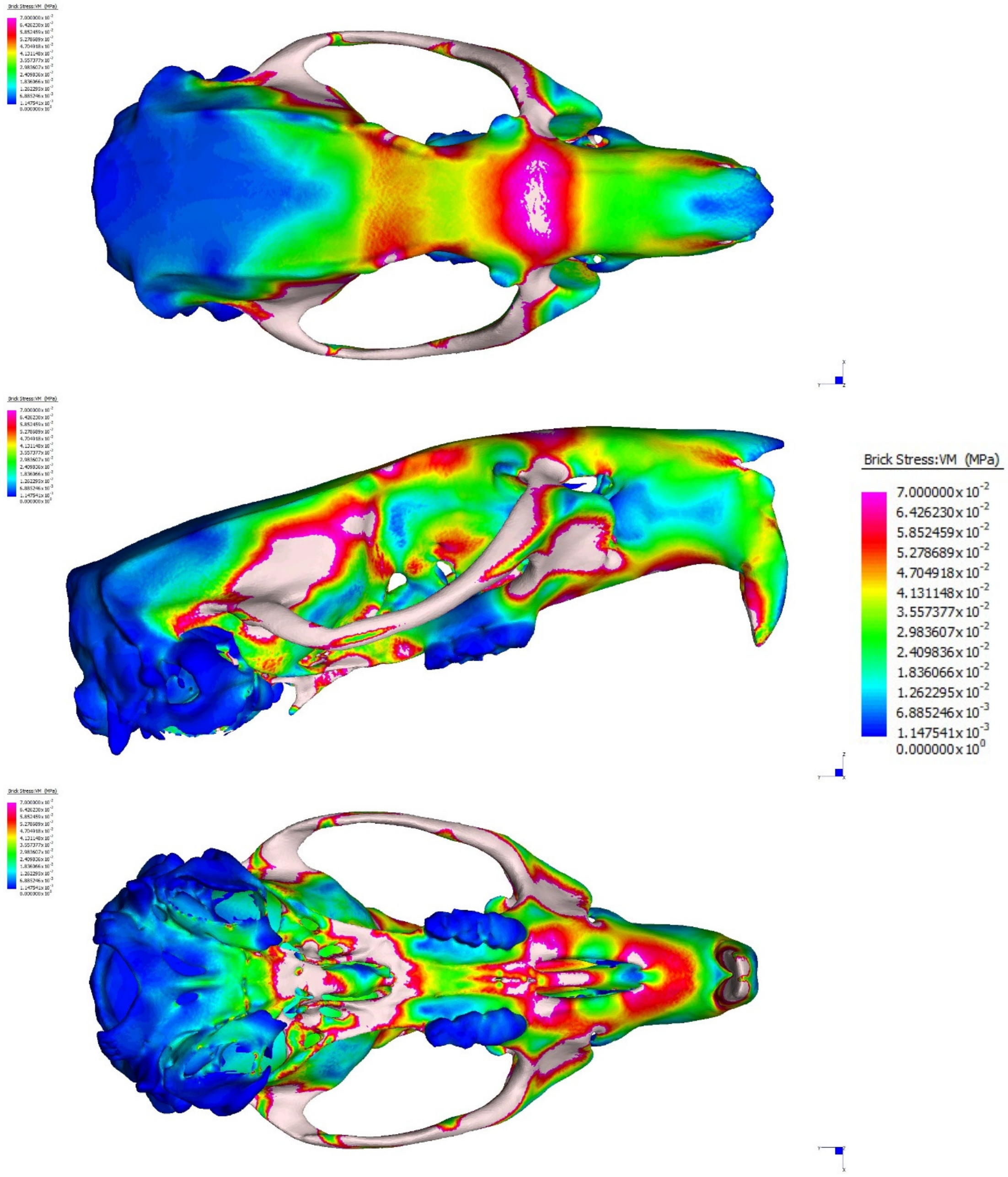
Brick Stress:VM (MPa)
7.000000x10^-2
6.426230x10^-2
5.852459x10^-2
5.278689x10^-2
4.704918x10^-2
4.131148x10^-2
3.557377x10^-2
2.983607x10^-2
2.409836x10^-2
1.836066x10^-2
1.262295x10^-2
6.885246x10^-3
1.147541x10^-3
0.000000x10^0

G2A6

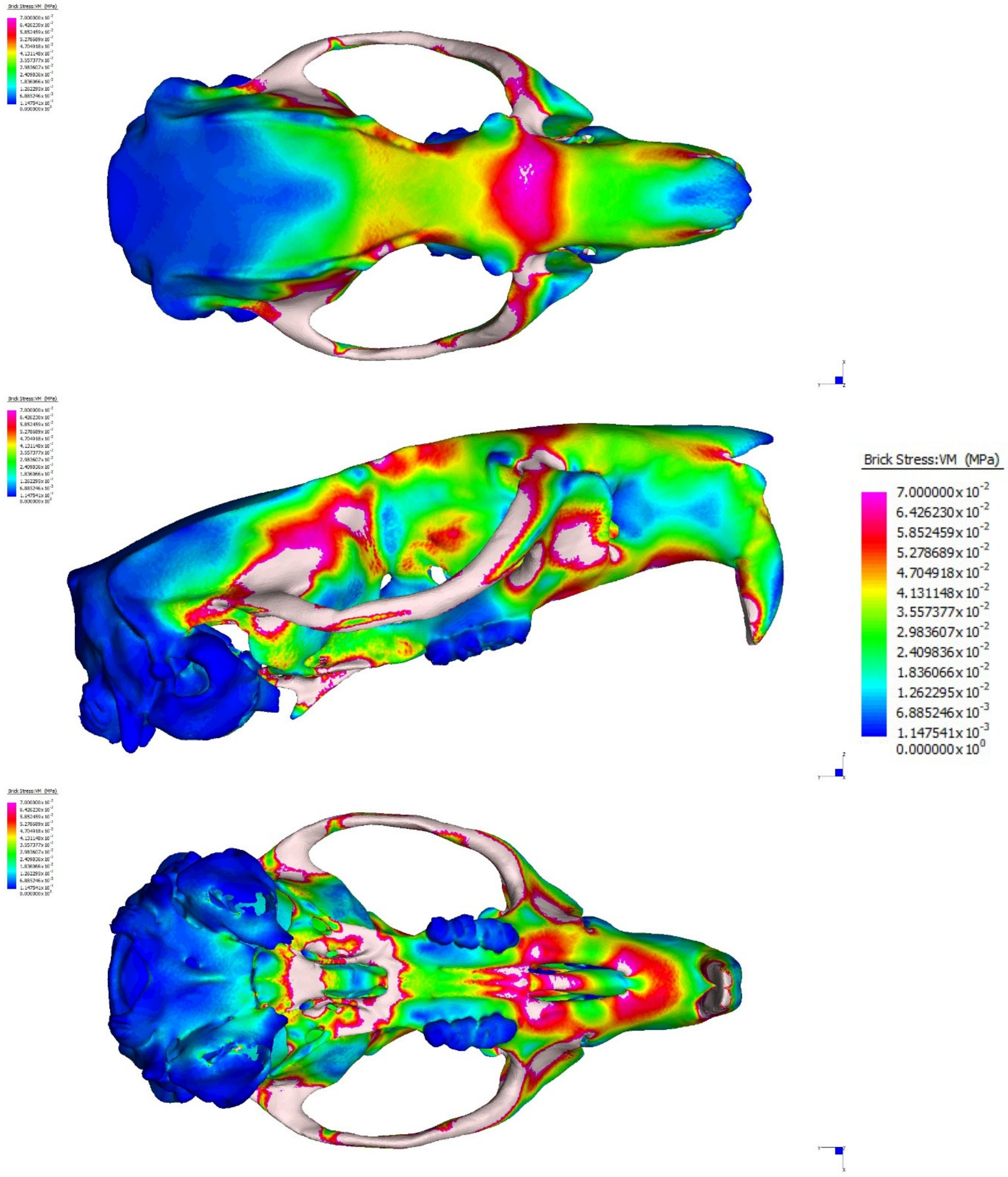

Brick Stress:VM (MPa)
7.000000x10^-2
6.426230x10^-2
5.852459x10^-2
5.278689x10^-2
4.704918x10^-2
4.131148x10^-2
3.557377x10^-2
2.983607x10^-2
2.409836x10^-2
1.836066x10^-2
1.262295x10^-2
6.885246x10^-3
1.147541x10^-3
0.000000x10^0

G2A7

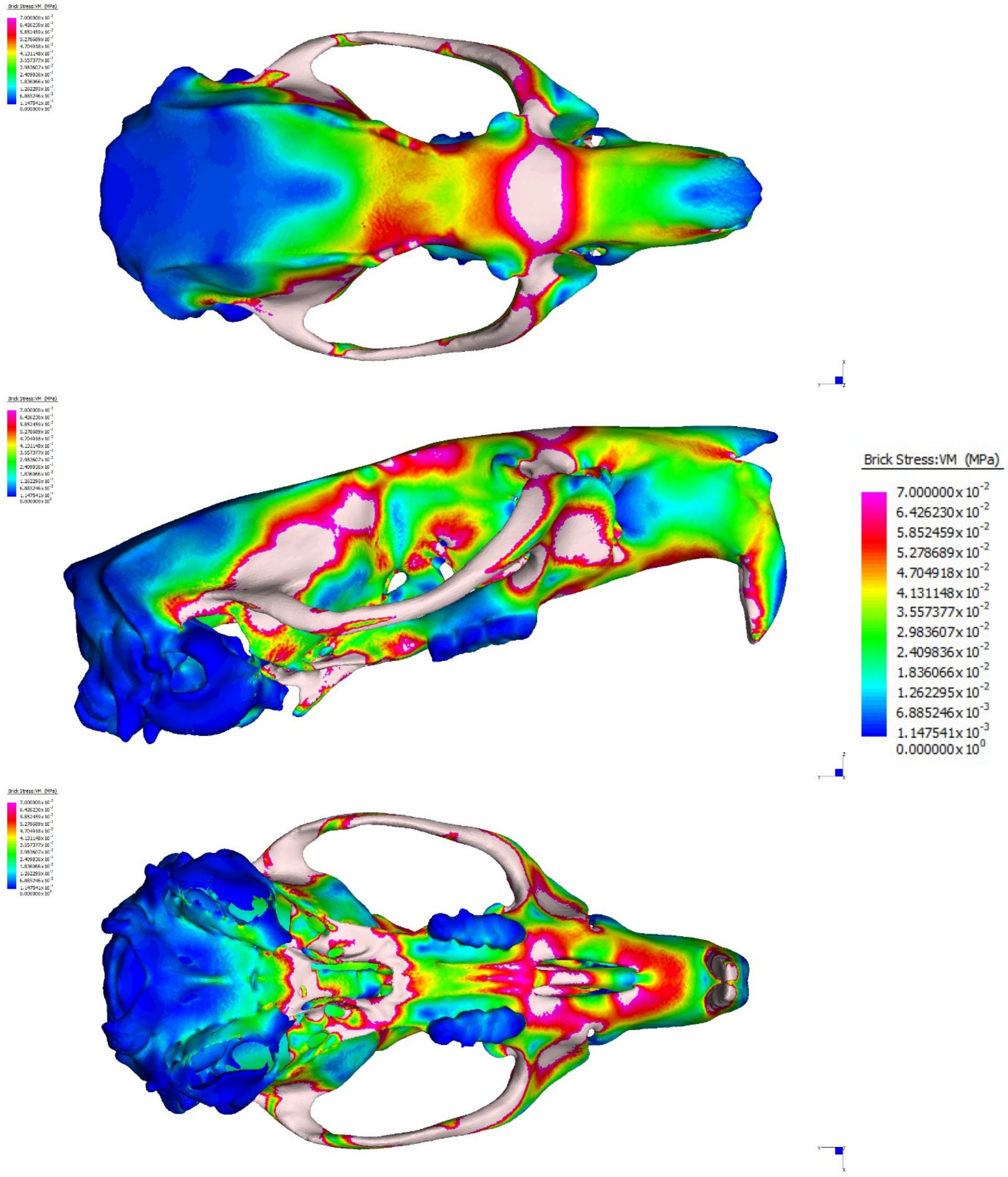
Brick Stress:VM (MPa)
7.000000x10^-2
6.426230x10^-2
5.852459x10^-2
5.278689x10^-2
4.704918x10^-2
4.131148x10^-2
3.557377x10^-2
2.983607x10^-2
2.409836x10^-2
1.836066x10^-2
1.262295x10^-2
6.885246x10^-3
1.147541x10^-3
0.000000x10^0

G2A8

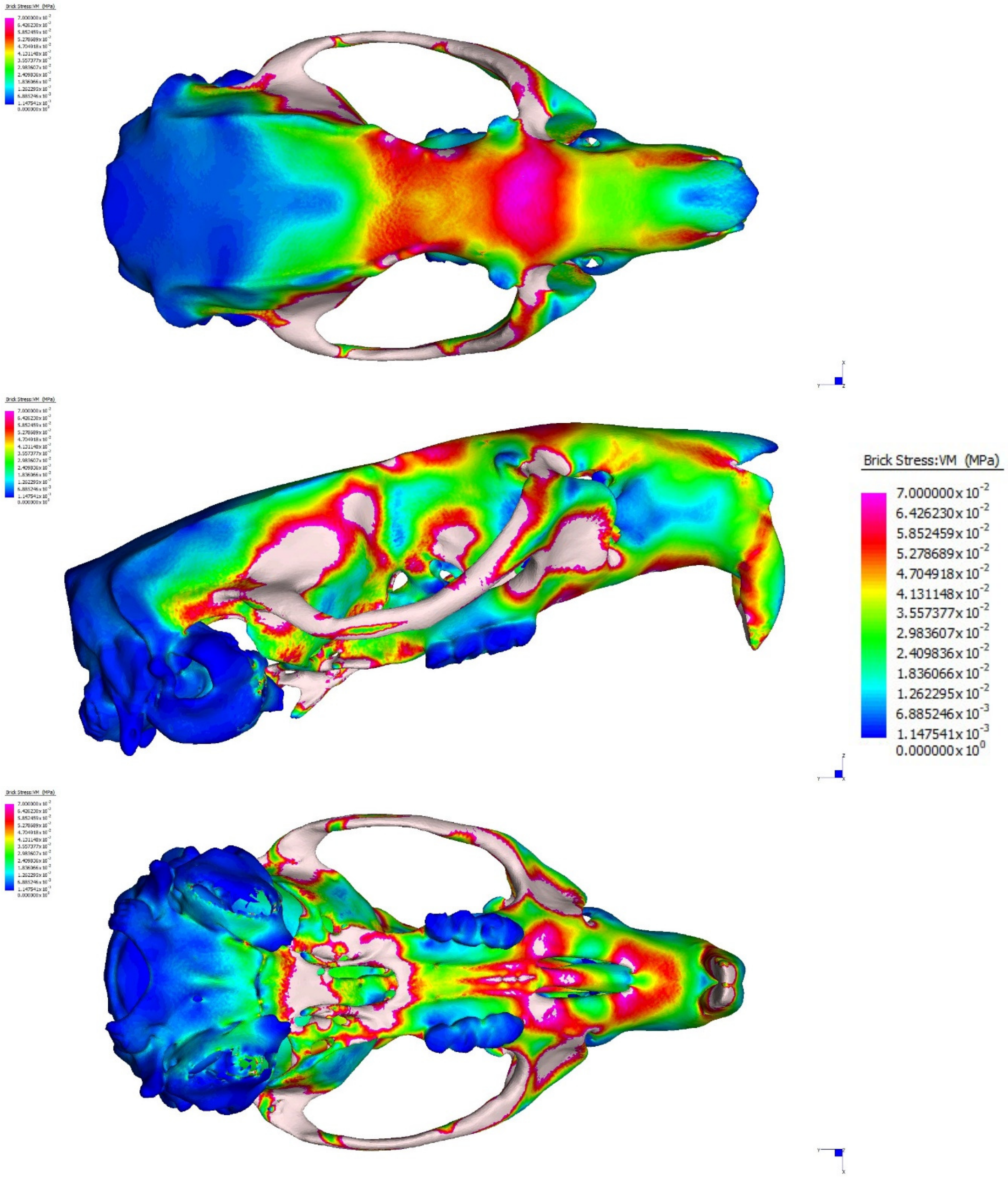
Brick Stress:VM (MPa)
7.000000x10^-2
6.426230x10^-2
5.852459x10^-2
5.278689x10^-2
4.704918x10^-2
4.131148x10^-2
3.557377x10^-2
2.983607x10^-2
2.409836x10^-2
1.836066x10^-2
1.262295x10^-2
6.885246x10^-3
1.147541x10^-3
0.000000x10^0

G2A9

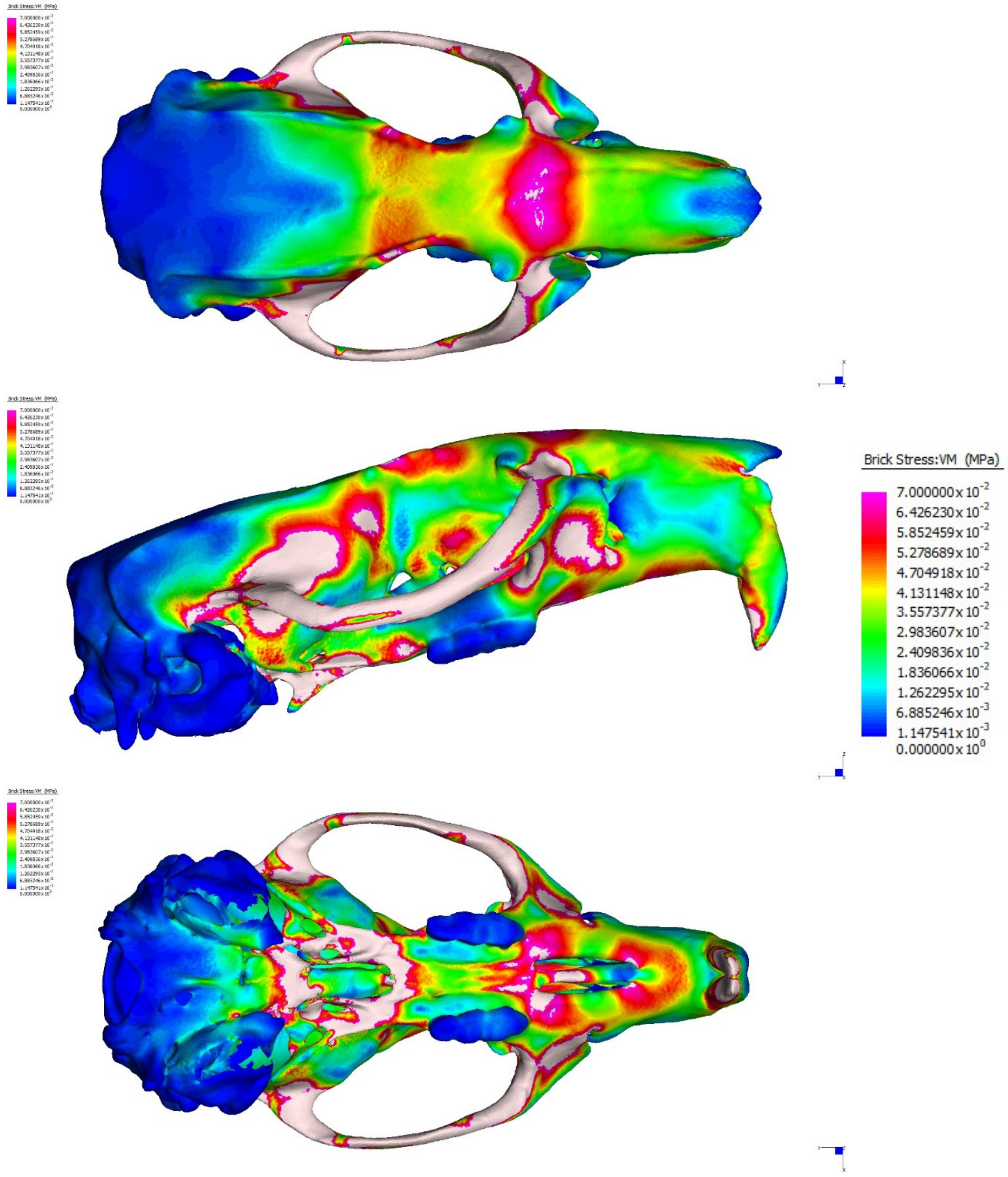
Brick Stress:VM (MPa)
7.000000 x 10^-2
6.426230 x 10^-2
5.852459 x 10^-2
5.278689 x 10^-2
4.704918 x 10^-2
4.131148 x 10^-2
3.557377 x 10^-2
2.983607 x 10^-2
2.409836 x 10^-2
1.836066 x 10^-2
1.262295 x 10^-2
6.885246 x 10^-3
1.147541 x 10^-3
0.000000 x 10^0

G2A10

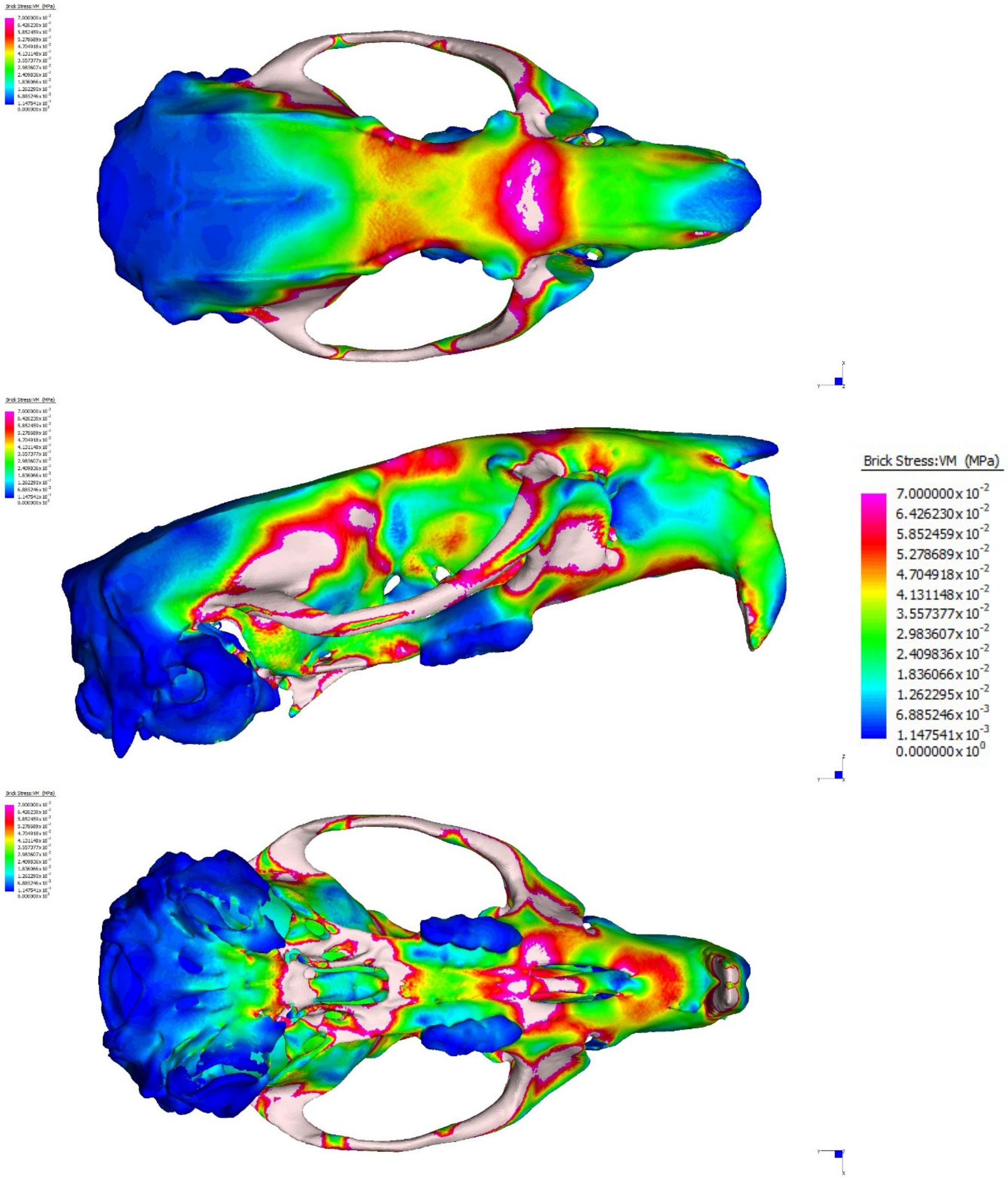
Brick Stress:VM (MPa)
7.000000x10^-2
6.426230x10^-2
5.852459x10^-2
5.278689x10^-2
4.704918x10^-2
4.131148x10^-2
3.557377x10^-2
2.983607x10^-2
2.409836x10^-2
1.836066x10^-2
1.262295x10^-2
6.885246x10^-3
1.147541x10^-3
0.000000x10^0

G3A1

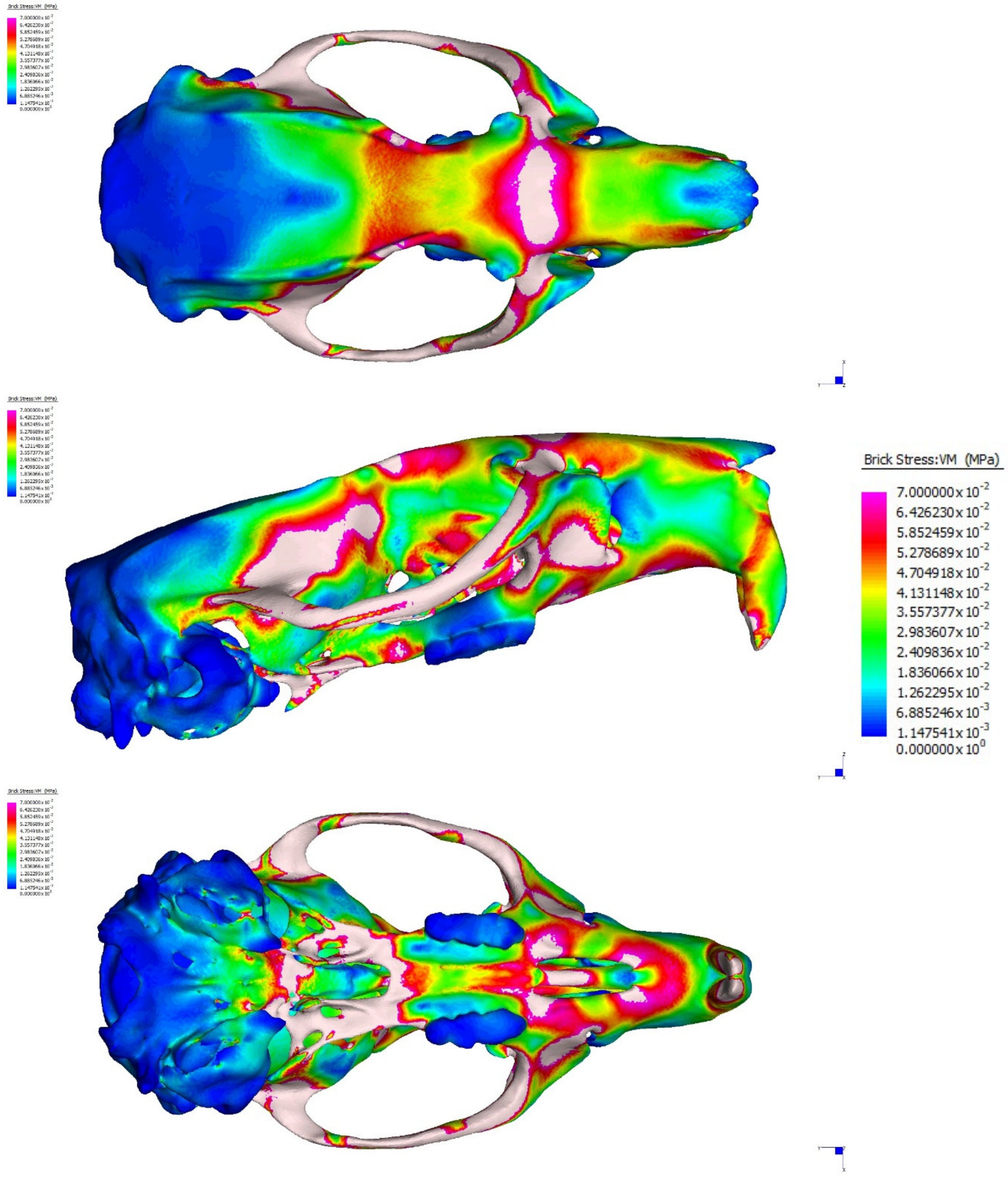
Brick Stress:VM (MPa)
7.000000x10^-2
6.426230x10^-2
5.852459x10^-2
5.278689x10^-2
4.704918x10^-2
4.131148x10^-2
3.557377x10^-2
2.983607x10^-2
2.409836x10^-2
1.836066x10^-2
1.262295x10^-2
6.885246x10^-3
1.147541x10^-3
0.000000x10^0

G3A2

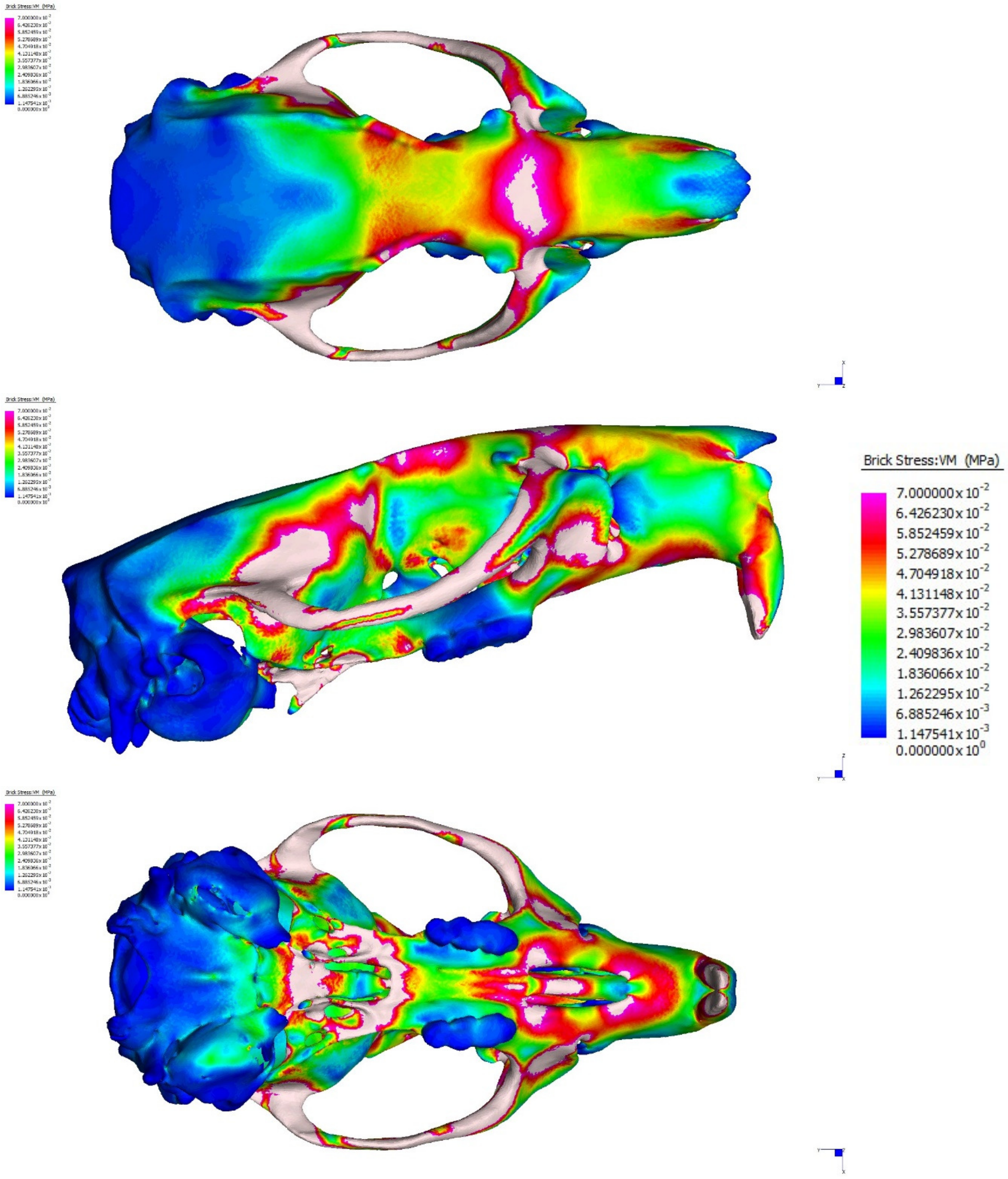

Brick Stress:VM (MPa)
7.000000x10^-2
6.426230x10^-2
5.852459x10^-2
5.278689x10^-2
4.704918x10^-2
4.131148x10^-2
3.557377x10^-2
2.983607x10^-2
2.409836x10^-2
1.836066x10^-2
1.262295x10^-2
6.885246x10^-3
1.147541x10^-3
0.000000x10^0

G3A3

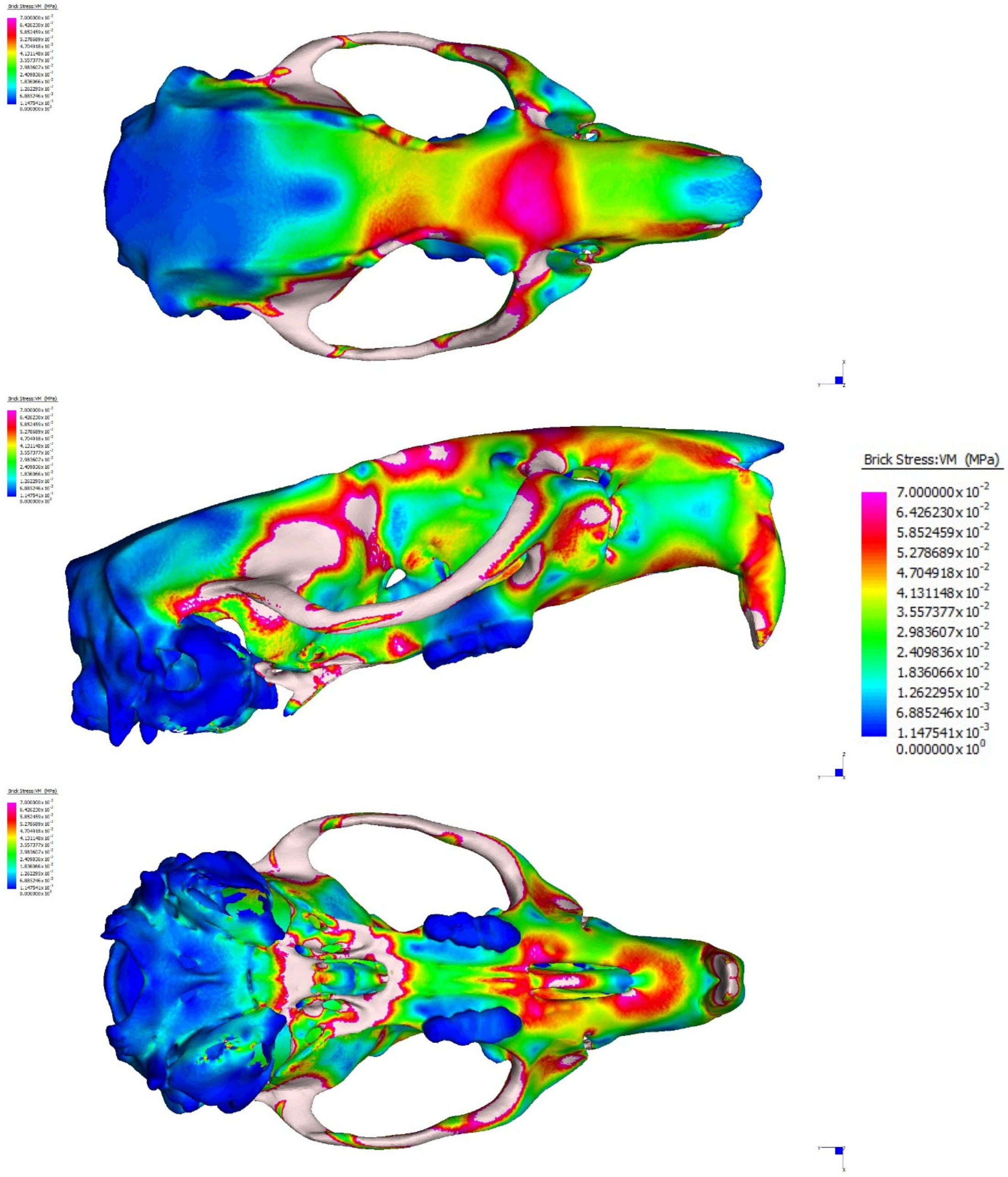
Brick Stress:VM (MPa)
7.000000x10^-2
6.426230x10^-2
5.852459x10^-2
5.278689x10^-2
4.704918x10^-2
4.131148x10^-2
3.557377x10^-2
2.983607x10^-2
2.409836x10^-2
1.836066x10^-2
1.262295x10^-2
6.885246x10^-3
1.147541x10^-3
0.000000x10^0

G3A4

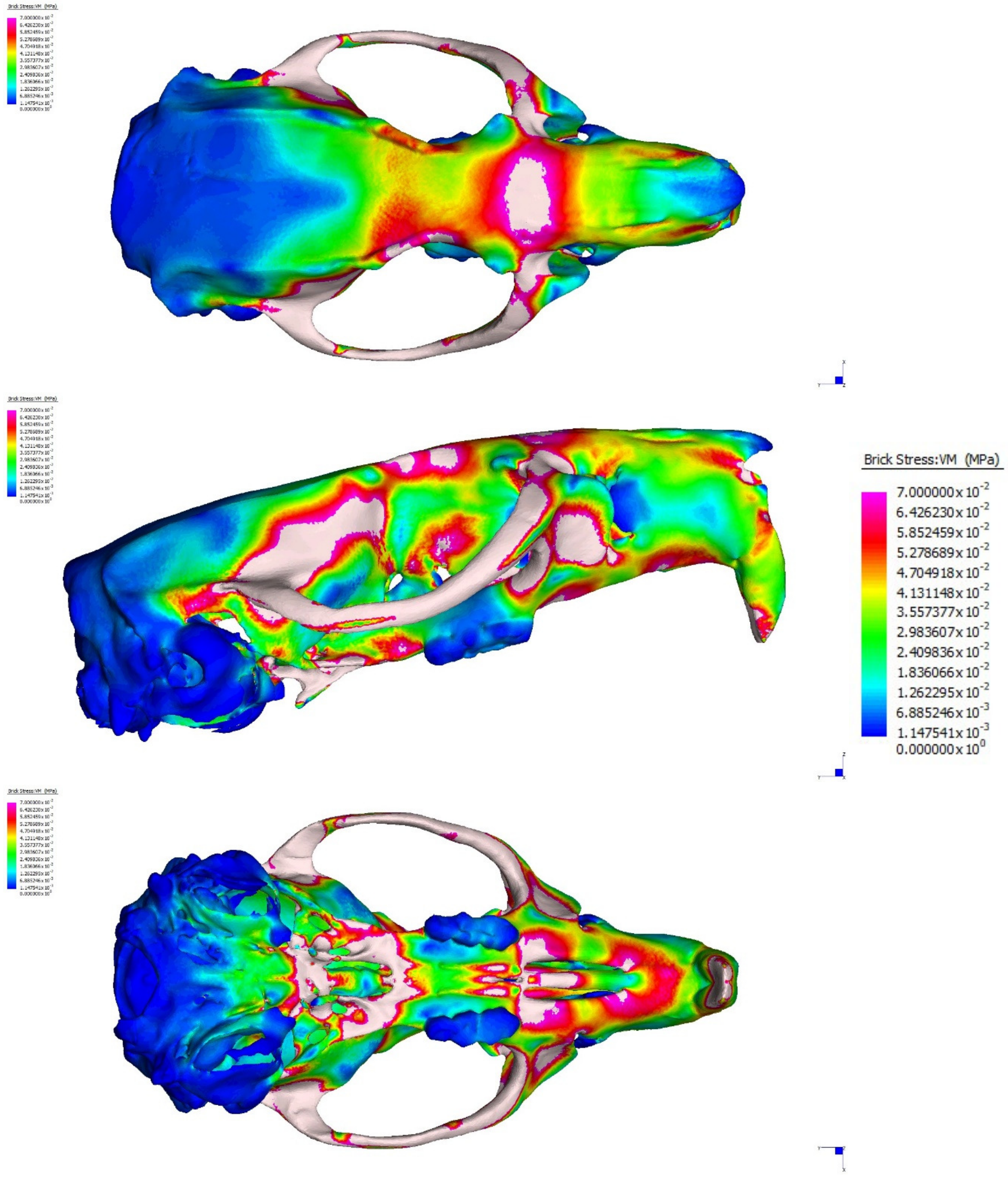

Brick Stress:VM (MPa)
7.000000×10^{-2}
6.426230×10^{-2}
5.852459×10^{-2}
5.278689×10^{-2}
4.704918×10^{-2}
4.131148×10^{-2}
3.557377×10^{-2}
2.983607×10^{-2}
2.409836×10^{-2}
1.836066×10^{-2}
1.262295×10^{-2}
6.885246×10^{-3}
1.147541×10^{-3}
0.000000×10^{0}

G3A5

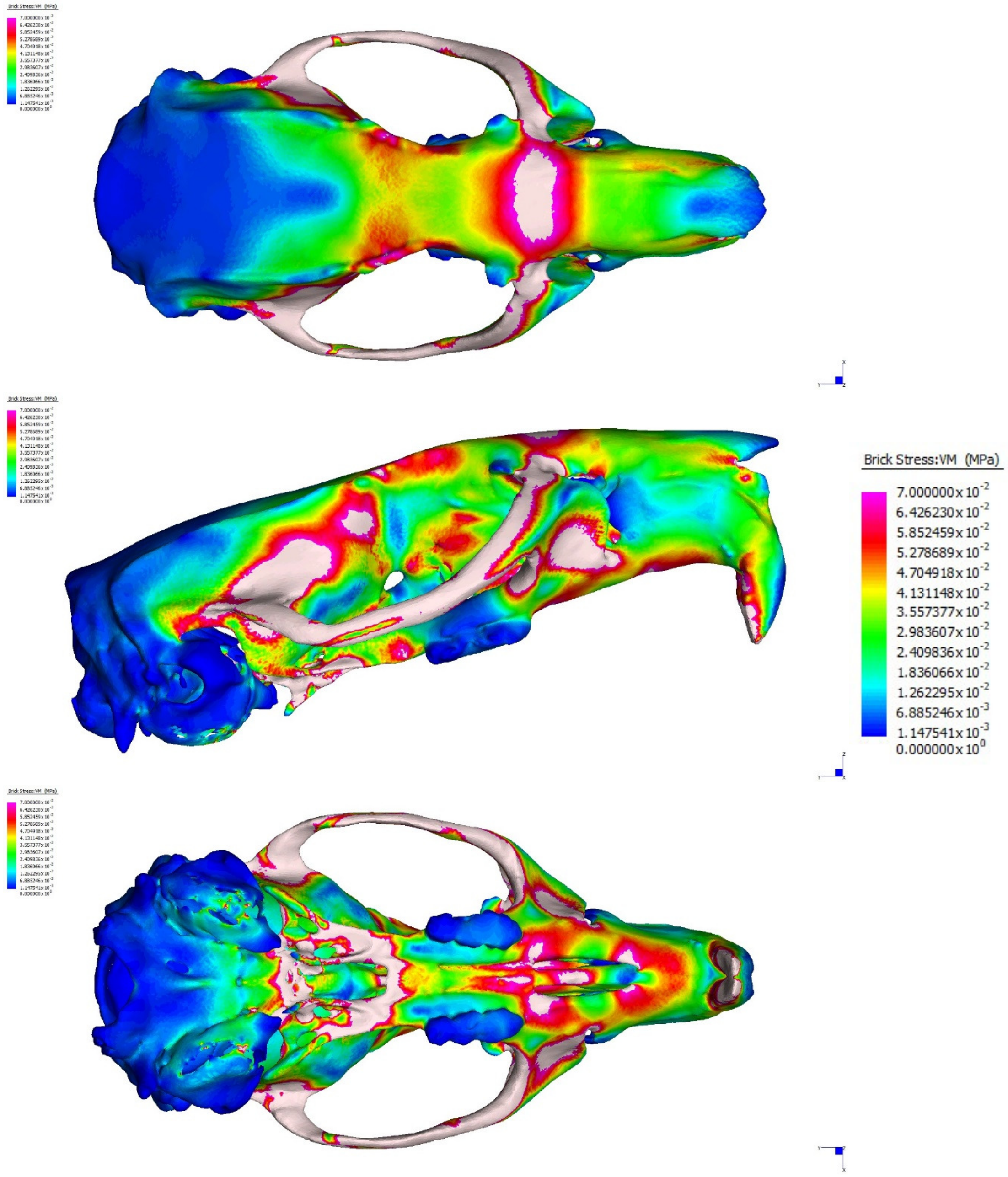
Brick Stress:VM (MPa)
7.000000x10^-2
6.426230x10^-2
5.852459x10^-2
5.278689x10^-2
4.704918x10^-2
4.131148x10^-2
3.557377x10^-2
2.983607x10^-2
2.409836x10^-2
1.836066x10^-2
1.262295x10^-2
6.885246x10^-3
1.147541x10^-3
0.000000x10^0

G3A6

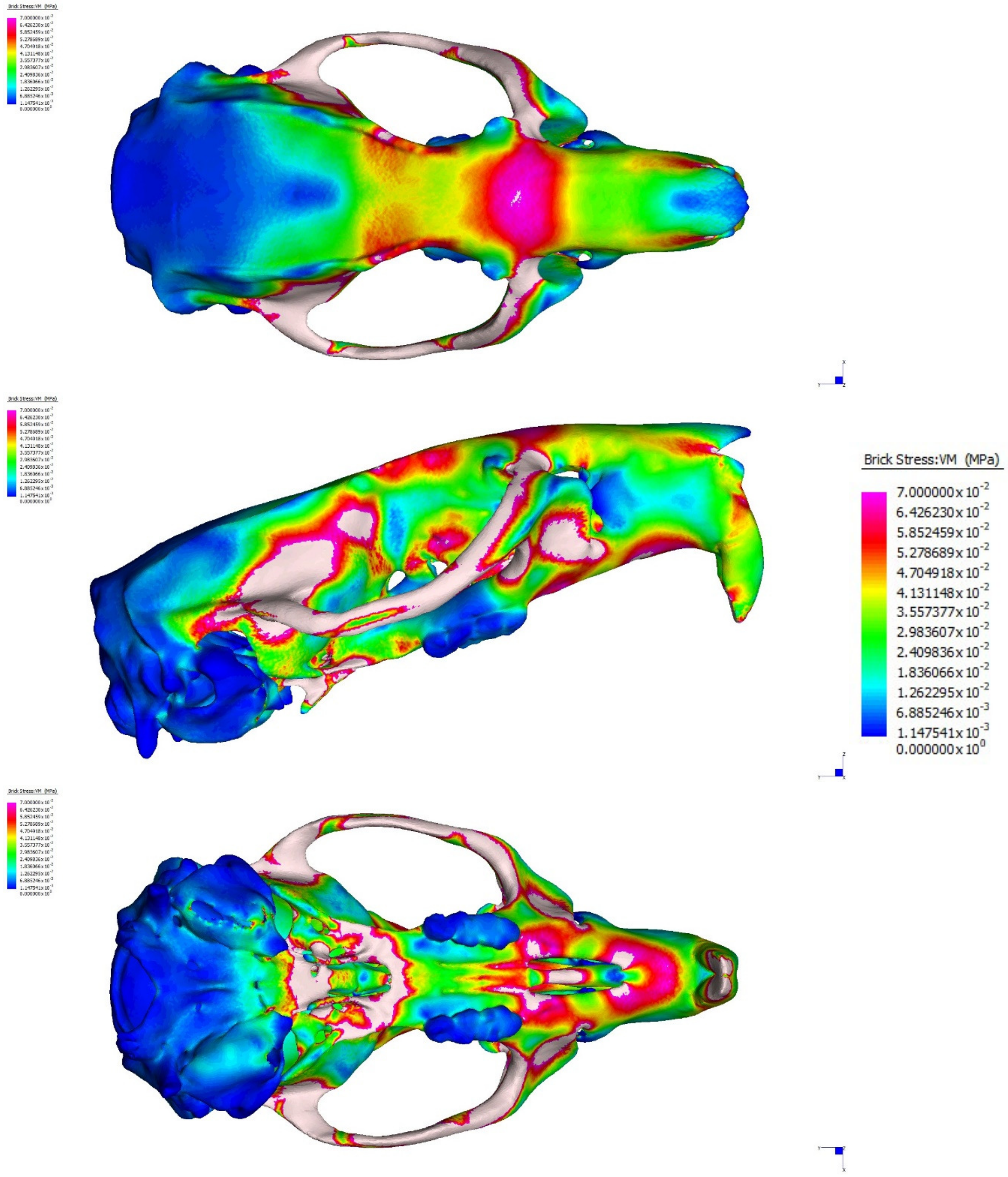

Brick Stress:VM (MPa)
7.000000x10^-2
6.426230x10^-2
5.852459x10^-2
5.278689x10^-2
4.704918x10^-2
4.131148x10^-2
3.557377x10^-2
2.983607x10^-2
2.409836x10^-2
1.836066x10^-2
1.262295x10^-2
6.885246x10^-3
1.147541x10^-3
0.000000x10^0

G3A7

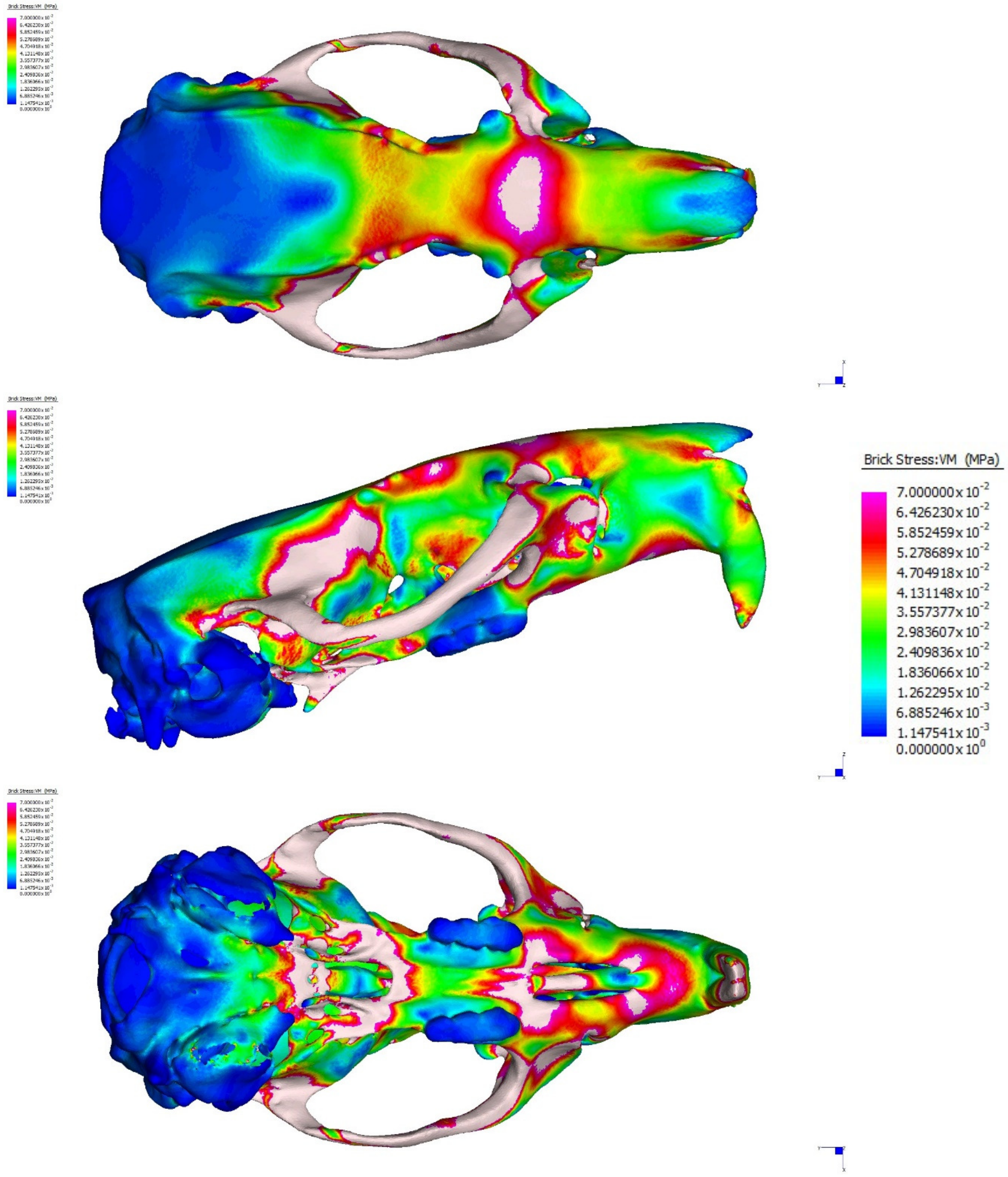
Brick Stress:VM (MPa)
7.000000x10^-2
6.426230x10^-2
5.852459x10^-2
5.278689x10^-2
4.704918x10^-2
4.131148x10^-2
3.557377x10^-2
2.983607x10^-2
2.409836x10^-2
1.836066x10^-2
1.262295x10^-2
6.885246x10^-3
1.147541x10^-3
0.000000x10^0

G3A8

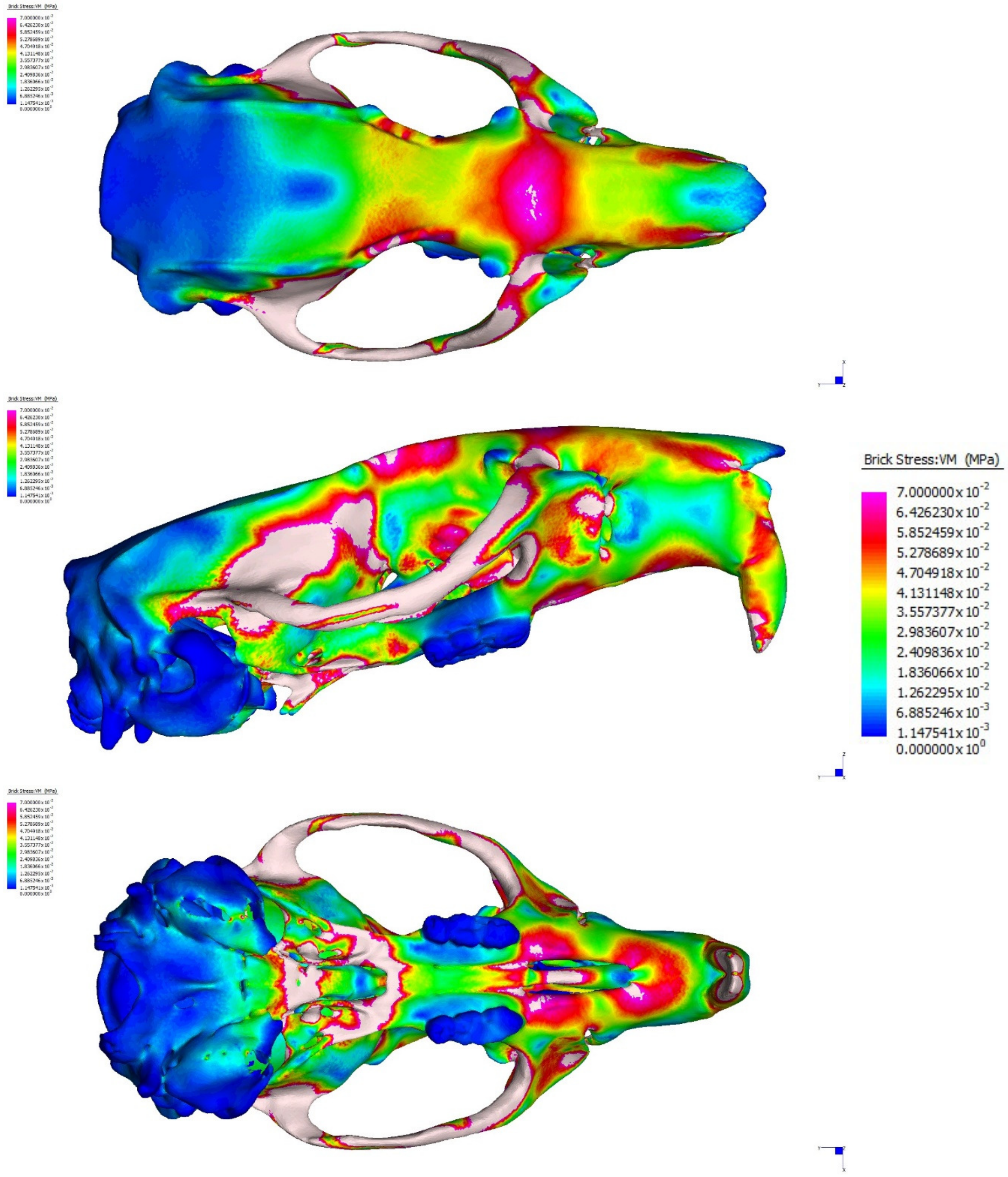

Brick Stress:VM (MPa)
7.000000x10^-2
6.426230x10^-2
5.852459x10^-2
5.278689x10^-2
4.704918x10^-2
4.131148x10^-2
3.557377x10^-2
2.983607x10^-2
2.409836x10^-2
1.836066x10^-2
1.262295x10^-2
6.885246x10^-3
1.147541x10^-3
0.000000x10^0

G3A9

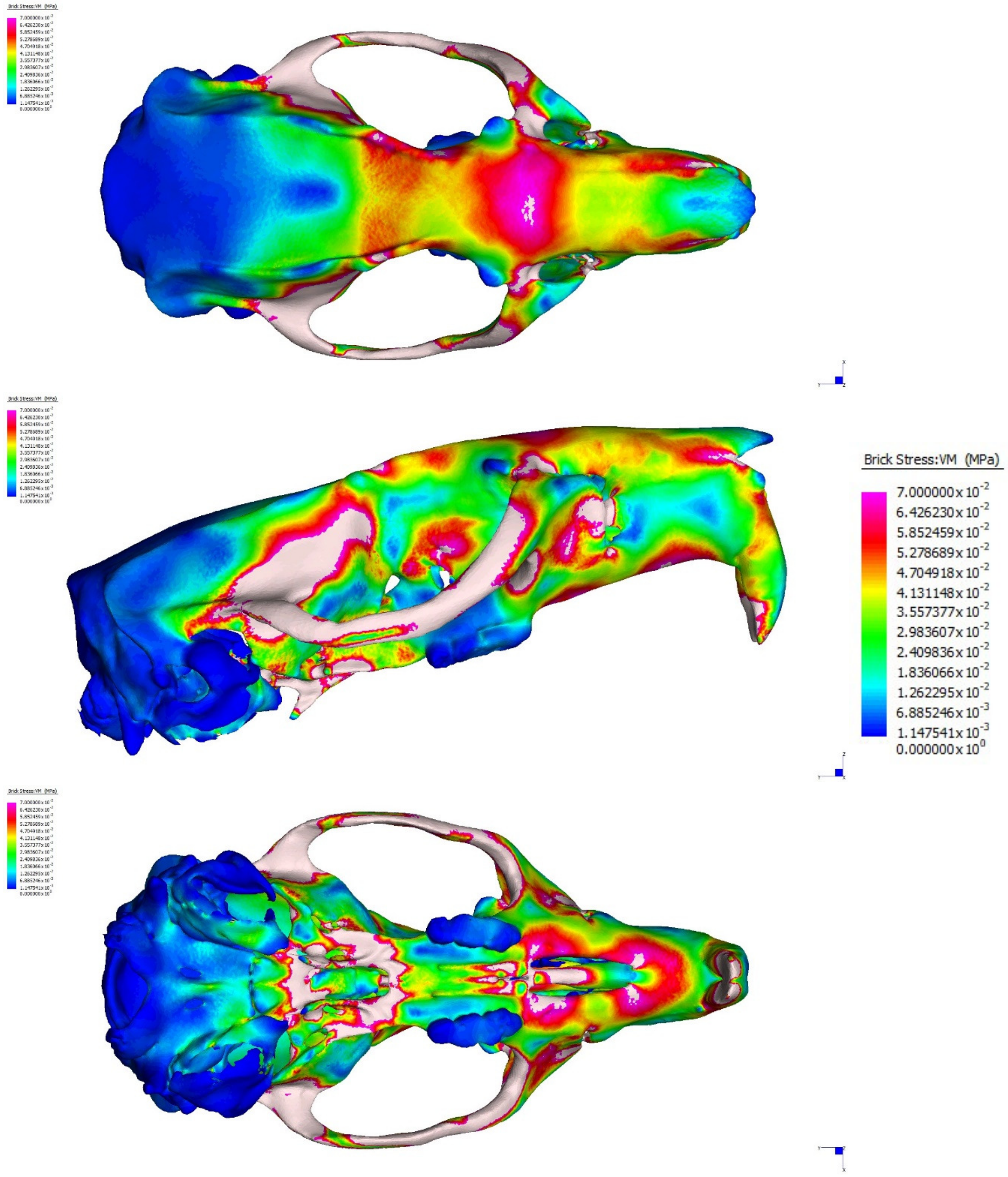
Brick Stress:VM (MPa)
7.000000 x 10^-2
6.426230 x 10^-2
5.852459 x 10^-2
5.278689 x 10^-2
4.704918 x 10^-2
4.131148 x 10^-2
3.557377 x 10^-2
2.983607 x 10^-2
2.409836 x 10^-2
1.836066 x 10^-2
1.262295 x 10^-2
6.885246 x 10^-3
1.147541 x 10^-3
0.000000 x 10^0

G3A10

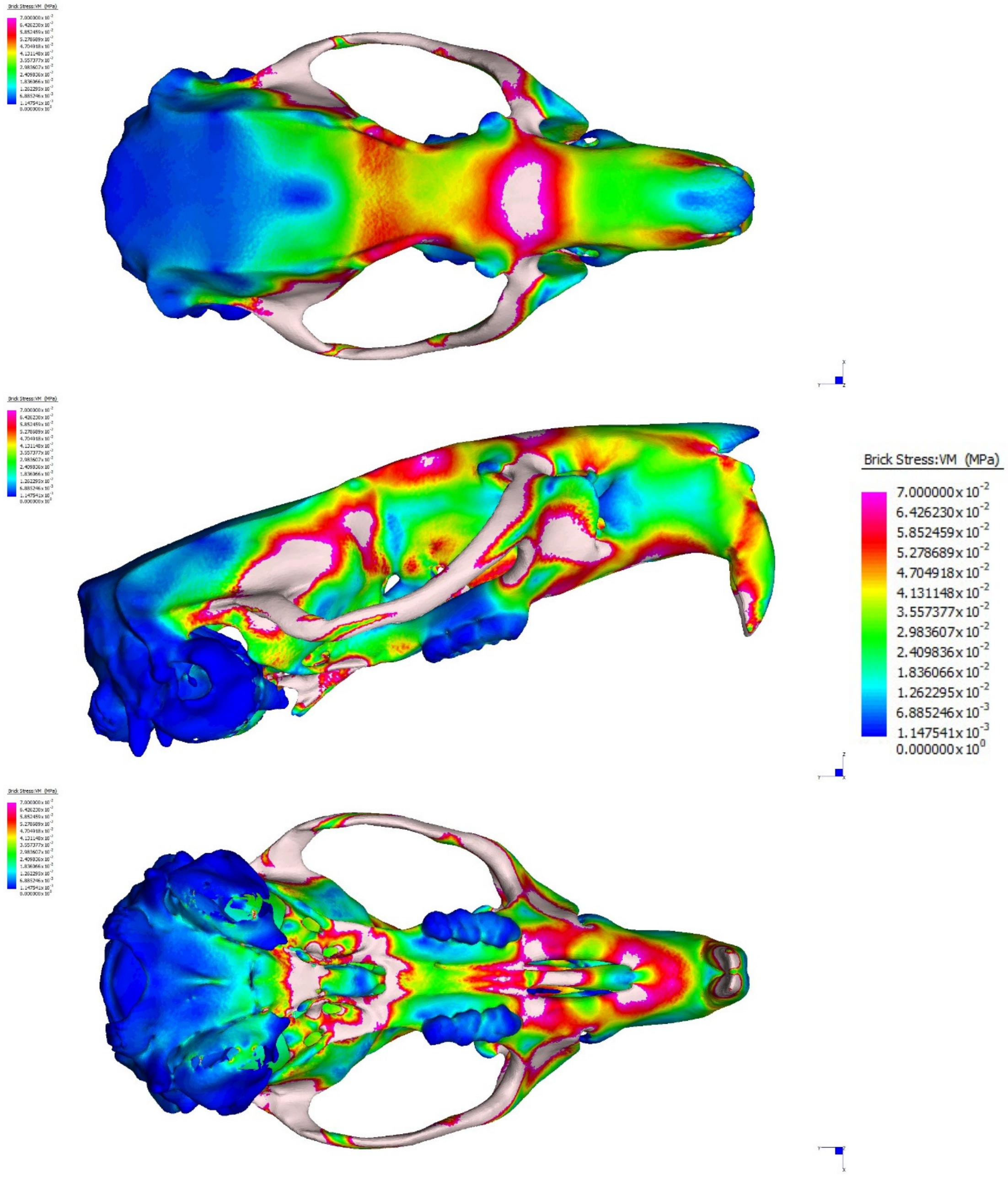
Brick Stress:VM (MPa)
7.000000x10^-2
6.426230x10^-2
5.852459x10^-2
5.278689x10^-2
4.704918x10^-2
4.131148x10^-2
3.557377x10^-2
2.983607x10^-2
2.409836x10^-2
1.836066x10^-2
1.262295x10^-2
6.885246x10^-3
1.147541x10^-3
0.000000x10^0

G4A1

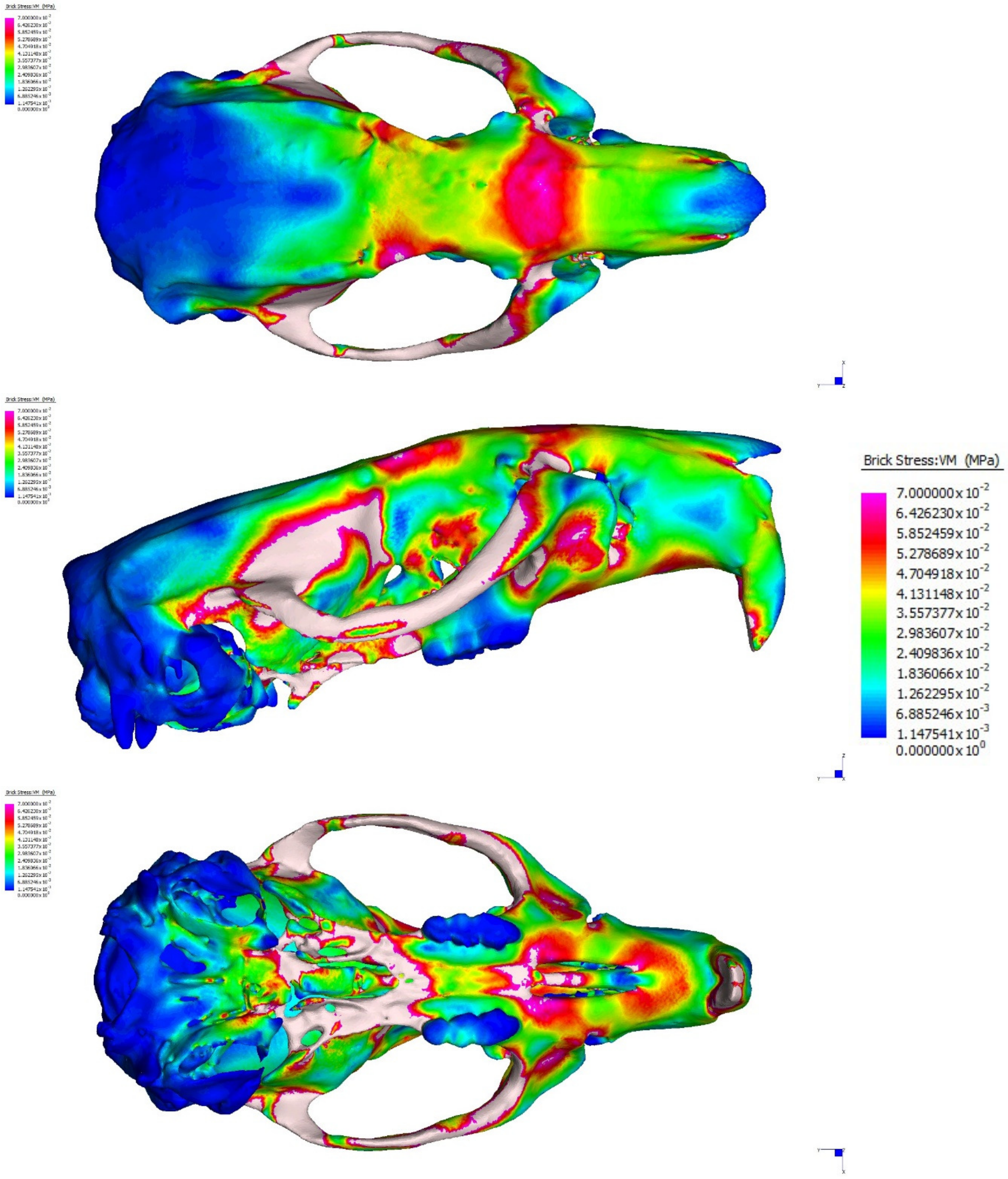
Brick Stress:VM (MPa)
7.000000×10^{-2}
6.426230×10^{-2}
5.852459×10^{-2}
5.278689×10^{-2}
4.704918×10^{-2}
4.131148×10^{-2}
3.557377×10^{-2}
2.983607×10^{-2}
2.409836×10^{-2}
1.836066×10^{-2}
1.262295×10^{-2}
6.885246×10^{-3}
1.147541×10^{-3}
0.000000×10^{0}

G4A3

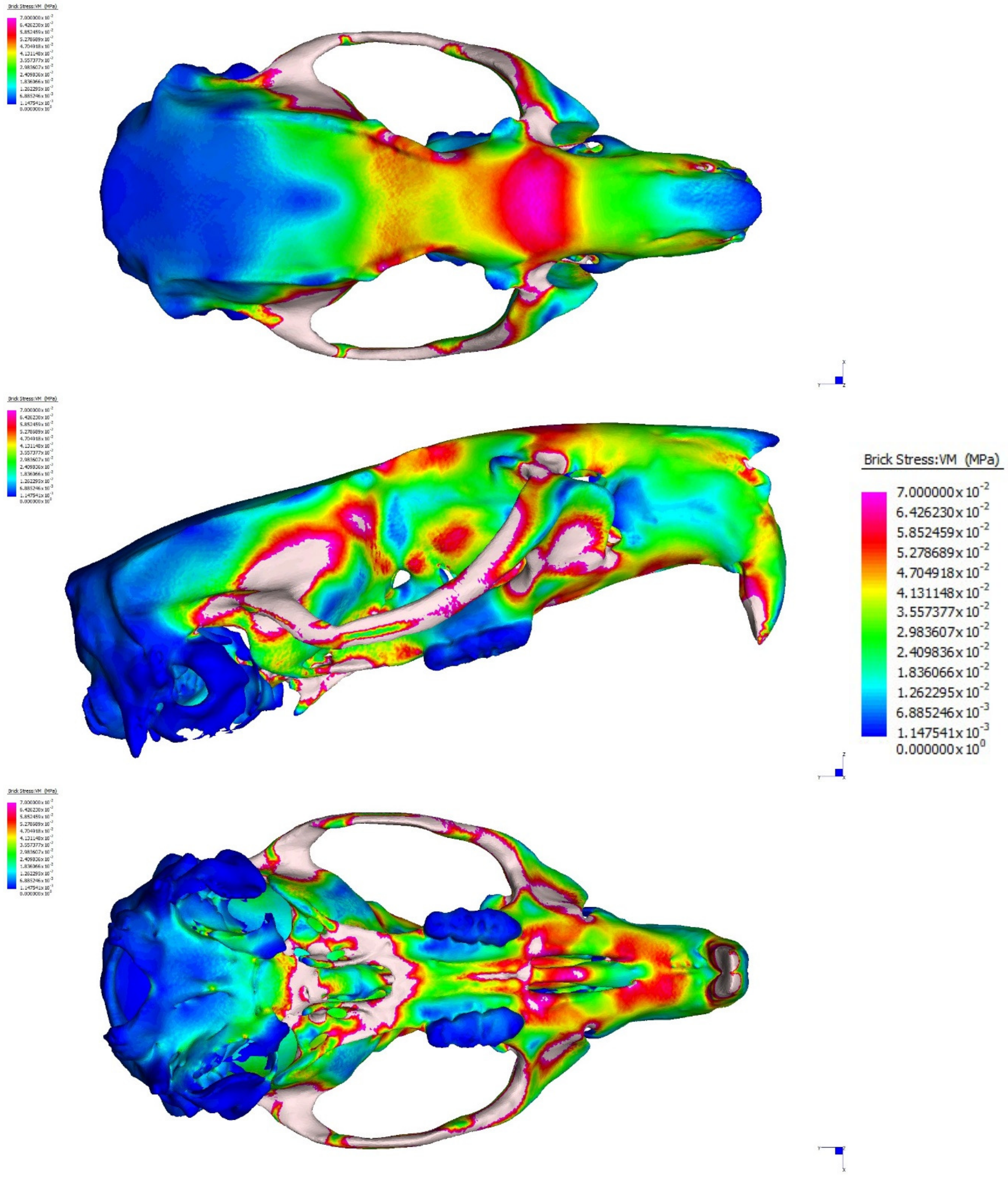

Brick Stress:VM (MPa)
7.000000×10^{-2}
6.426230×10^{-2}
5.852459×10^{-2}
5.278689×10^{-2}
4.704918×10^{-2}
4.131148×10^{-2}
3.557377×10^{-2}
2.983607×10^{-2}
2.409836×10^{-2}
1.836066×10^{-2}
1.262295×10^{-2}
6.885246×10^{-3}
1.147541×10^{-3}
0.000000×10^{0}

G4A4

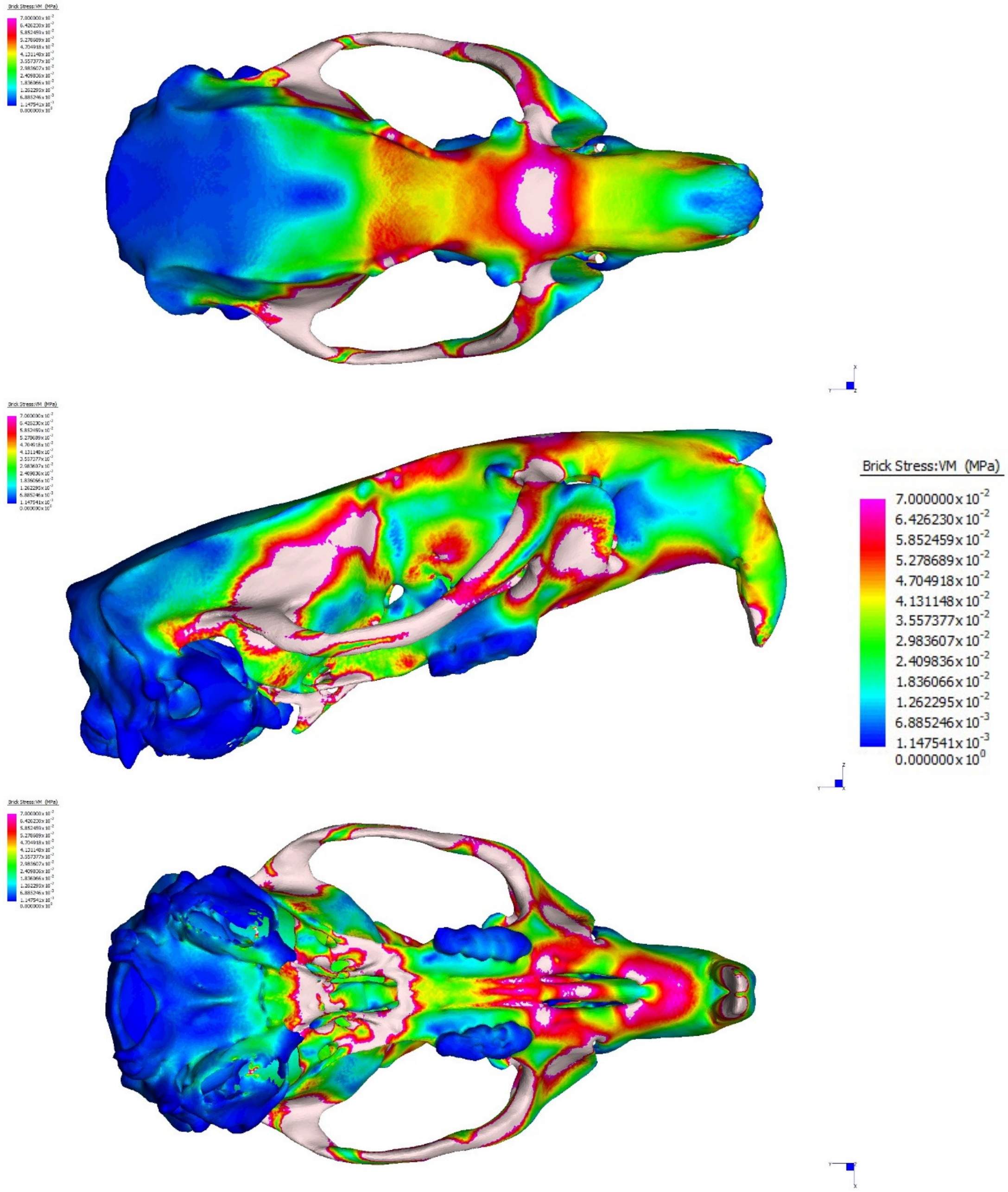
Brick Stress:VM (MPa)
7.000000x10^-2
6.426230x10^-2
5.852459x10^-2
5.278689x10^-2
4.704918x10^-2
4.131148x10^-2
3.557377x10^-2
2.983607x10^-2
2.409836x10^-2
1.836066x10^-2
1.262295x10^-2
6.885246x10^-3
1.147541x10^-3
0.000000x10^0

G4A5

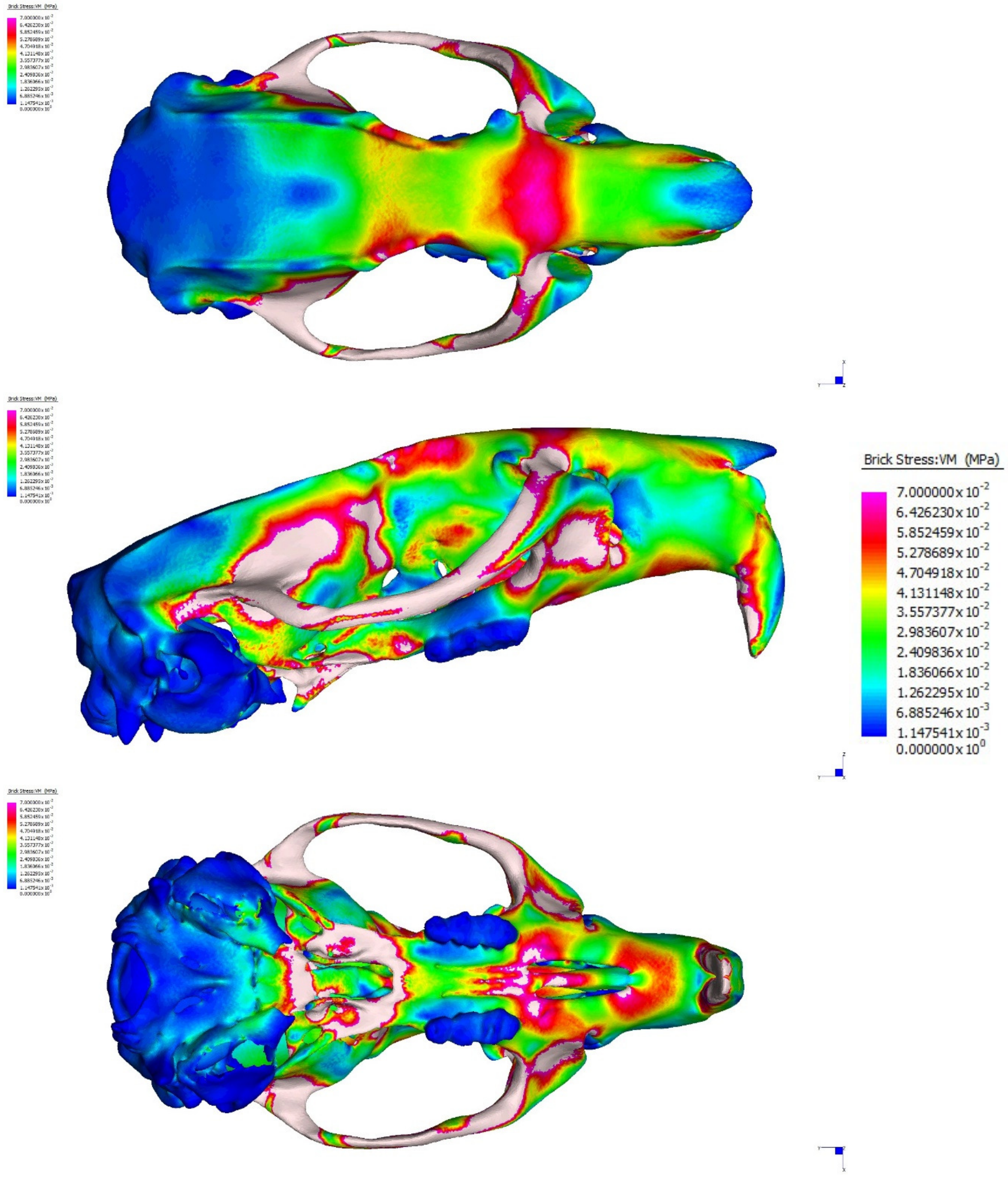
Brick Stress:VM (MPa)
7.000000x10-2
6.426230x10-2
5.852459x10-2
5.278689x10-2
4.704918x10-2
4.131148x10-2
3.557377x10-2
2.983607x10-2
2.409836x10-2
1.836066x10-2
1.262295x10-2
6.885246x10-3
1.147541x10-3
0.000000x100

G4A6

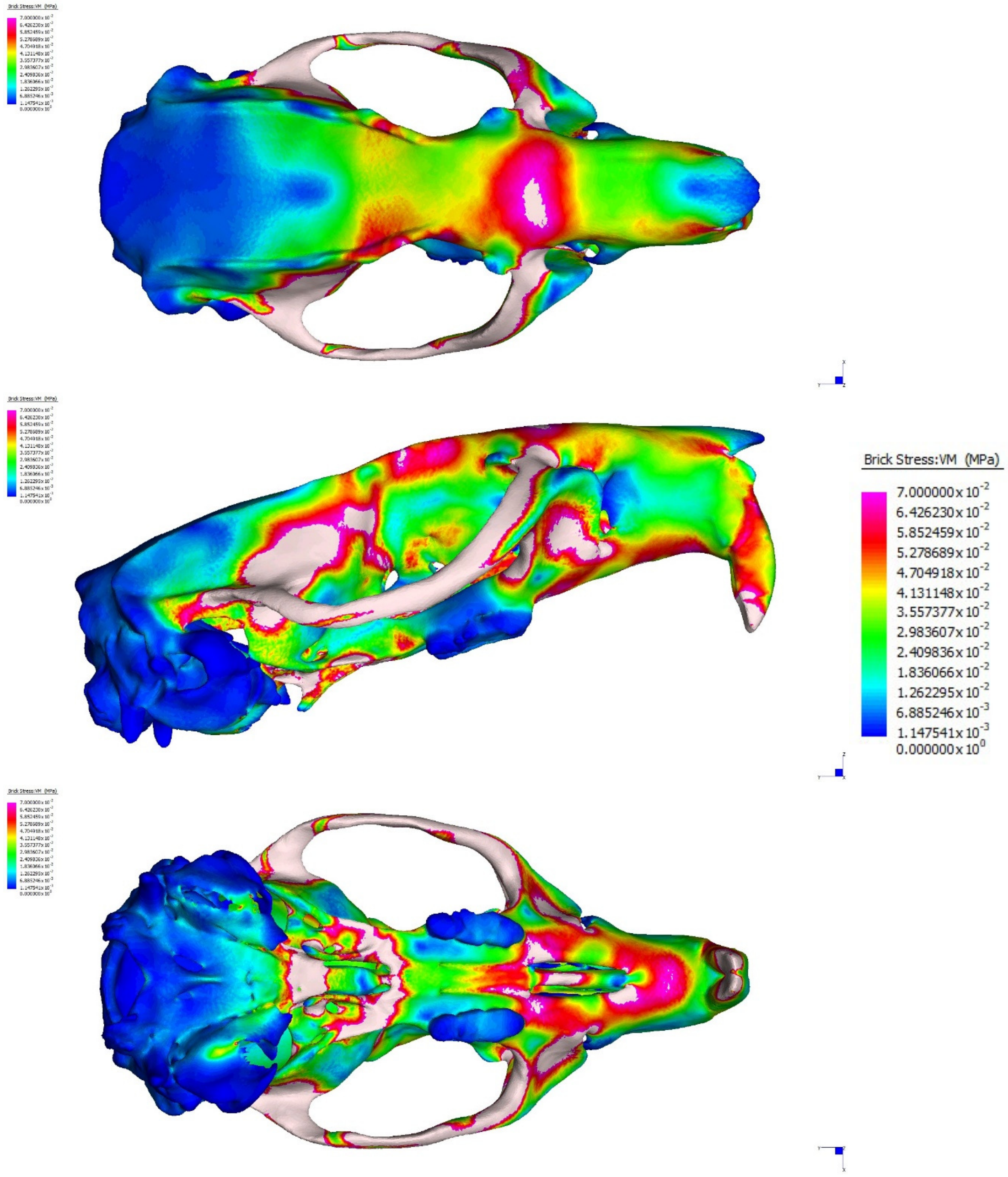
Brick Stress:VM (MPa)
7.000000x10^-2
6.426230x10^-2
5.852459x10^-2
5.278689x10^-2
4.704918x10^-2
4.131148x10^-2
3.557377x10^-2
2.983607x10^-2
2.409836x10^-2
1.836066x10^-2
1.262295x10^-2
6.885246x10^-3
1.147541x10^-3
0.000000x10^0

G4A7

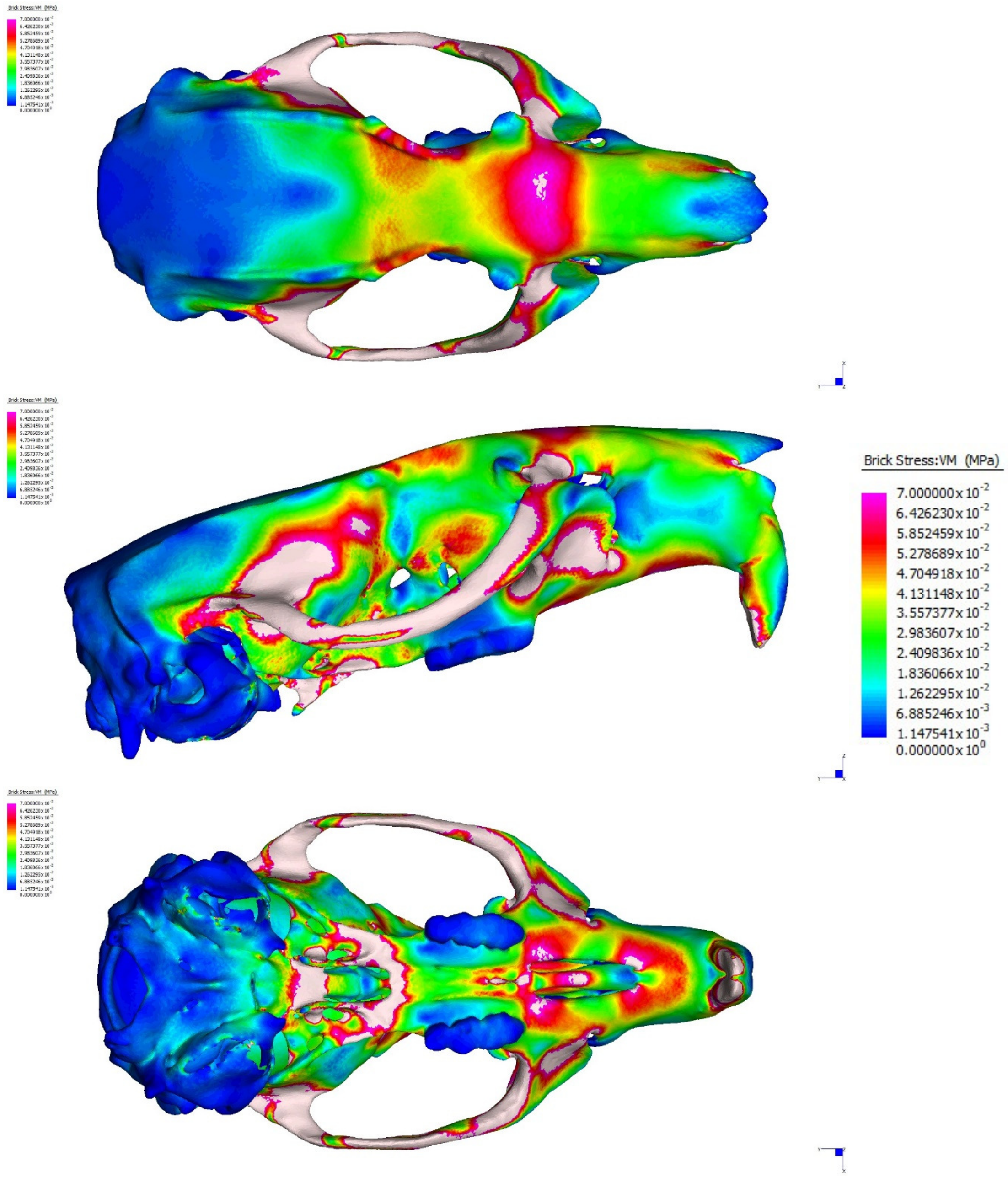
Brick Stress:VM (MPa)
7.000000x10^-2
6.426230x10^-2
5.852459x10^-2
5.278689x10^-2
4.704918x10^-2
4.131148x10^-2
3.557377x10^-2
2.983607x10^-2
2.409836x10^-2
1.836066x10^-2
1.262295x10^-2
6.885246x10^-3
1.147541x10^-3
0.000000x10^0

G4A8

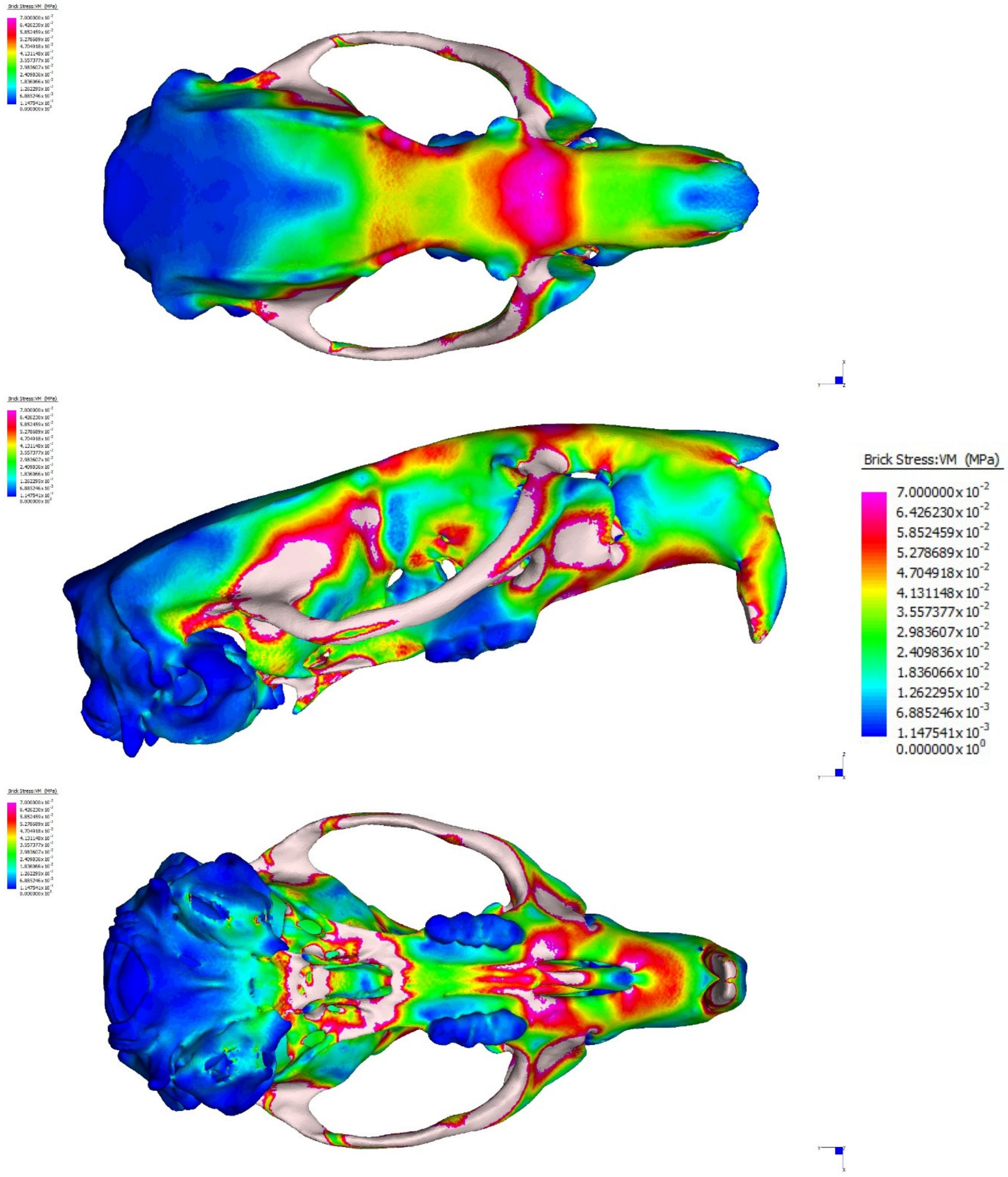
Brick Stress:VM (MPa)
7.000000x10^-2
6.426230x10^-2
5.852459x10^-2
5.278689x10^-2
4.704918x10^-2
4.131148x10^-2
3.557377x10^-2
2.983607x10^-2
2.409836x10^-2
1.836066x10^-2
1.262295x10^-2
6.885246x10^-3
1.147541x10^-3
0.000000x10^0

G4A9

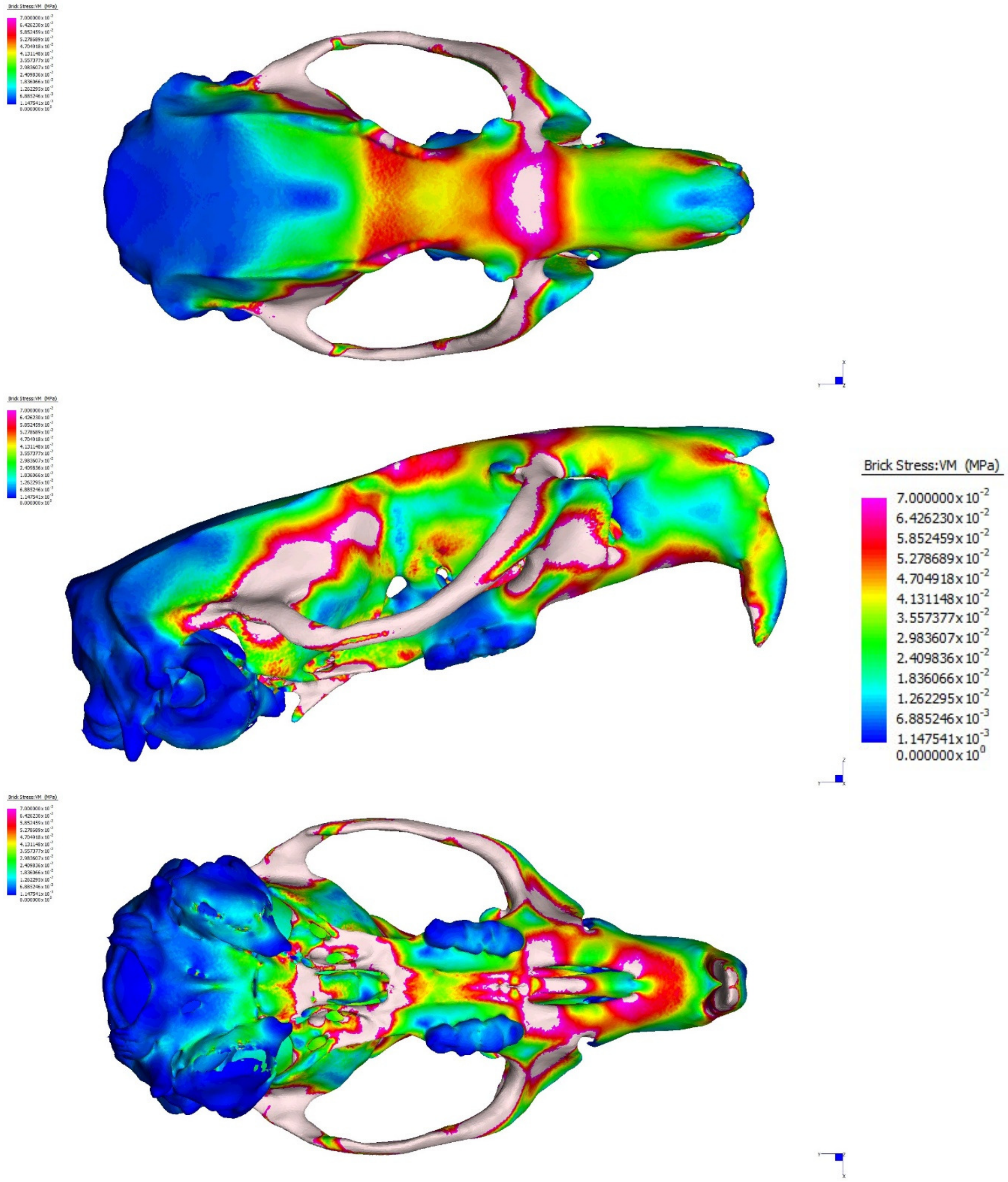
Brick Stress:VM (MPa)
7.000000×10^{-2}
6.426230×10^{-2}
5.852459×10^{-2}
5.278689×10^{-2}
4.704918×10^{-2}
4.131148×10^{-2}
3.557377×10^{-2}
2.983607×10^{-2}
2.409836×10^{-2}
1.836066×10^{-2}
1.262295×10^{-2}
6.885246×10^{-3}
1.147541×10^{-3}
0.000000×10^{0}

G4A10

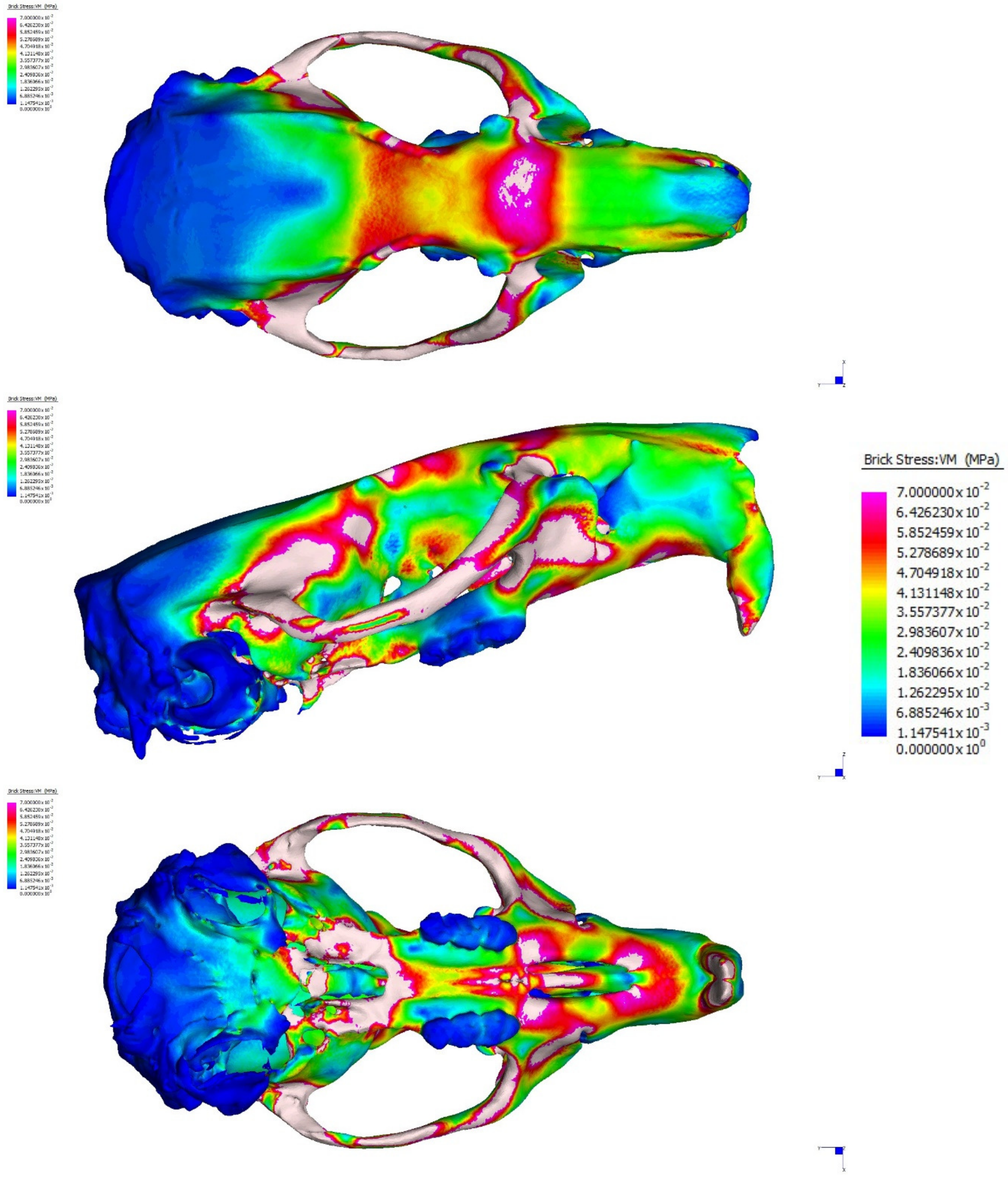
Brick Stress:VM (MPa)
7.000000x10^-2
6.426230x10^-2
5.852459x10^-2
5.278689x10^-2
4.704918x10^-2
4.131148x10^-2
3.557377x10^-2
2.983607x10^-2
2.409836x10^-2
1.836066x10^-2
1.262295x10^-2
6.885246x10^-3
1.147541x10^-3
0.000000x10^0

G4A11

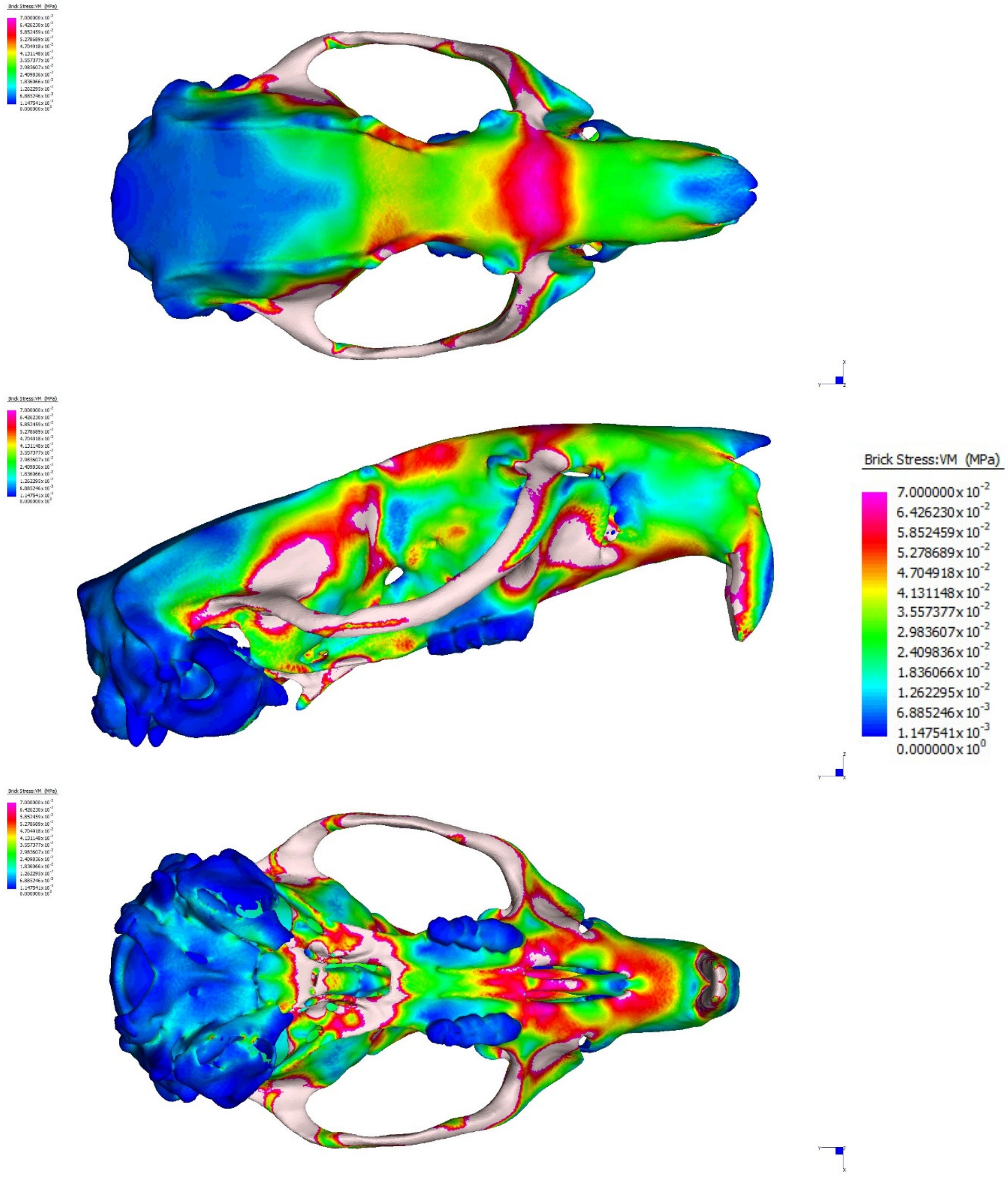
Brick Stress:VM (MPa)
7.000000×10^{-2}
6.426230×10^{-2}
5.852459×10^{-2}
5.278689×10^{-2}
4.704918×10^{-2}
4.131148×10^{-2}
3.557377×10^{-2}
2.983607×10^{-2}
2.409836×10^{-2}
1.836066×10^{-2}
1.262295×10^{-2}
6.885246×10^{-3}
1.147541×10^{-3}
0.000000×10^{0}
